# Differential anti-proliferative and apoptotic effects of lichen species on human prostate carcinoma cells

**DOI:** 10.1371/journal.pone.0238303

**Published:** 2020-09-30

**Authors:** Beyza Goncu, Ece Sevgi, Cagla Kizilarslan Hancer, Guzin Gokay, Nur Ozten

**Affiliations:** 1 Experimental Research Center, Bezmialem Vakif University, Istanbul, Turkey; 2 Department of Pharmaceutical Botany, Faculty of Pharmacy, BezmialemVakif University, Istanbul, Turkey; 3 Department of Pharmaceutical Toxicology, Faculty of Pharmacy, Bezmialem Vakif University, Istanbul, Turkey; Columbia University, UNITED STATES

## Abstract

Lichens are stable symbiotic associations between fungus and algae and/or cyanobacteria that have different biological activities. Around 60% of anti-cancer drugs are derived from natural resources including plants, fungi, sea creatures, and lichens. This project aims to identify the apoptotic effects and proliferative properties of extracts of *Bryoria capillaris (Ach*.*) Brodo* & *D*.*Hawksw*, *Cladonia fimbriata (L*.*) Fr*., *Evernia divaricata (L*.*) Ach*., *Hypogymnia tubulosa (Schaer*.*) Hav*., *Lobaria pulmonaria (L*.*) Hoffm*., and *Usnea florida (L*.*) Weber ex Wigg*. lichen species on prostate cancer cells. Lichen extracts were performed by ethanol, methanol, and acetone separately by using the Soxhlet apparatus and the effects of the extracts on cell viability, proliferation, and apoptosis were measured with the utilization of MTT, LDH assay, Annexin V assay, and Western Blot. Findings of our study revealed a positive correlation between the elevation of cell sensitivity and the increase in the treatment doses of the extract in that higher doses applied reverberate to higher cell sensitivity. A similar correlation was also identified between cell sensitivity elevation and the duration of the treatment. Evidence in our study have shown the existence of an anti-proliferative effect in the extracts of *Bryoria capillaris*, *Evernia divaricata (L*.*) Ach*., *Hypogymnia tubulosa (Schaer*.*) Hav*., *Lobaria pulmonaria (L*.*) Hoffm*., and *Usnea florida (L*.*) Weber ex Wigg*., while a similar effect was not observed in the extracts of *Cladonia fimbriata*. *Evernia divaricata* induced anti-proliferative and apoptotic effects in PC-3 cells, *which* induced apoptotic cell death by both extrinsic and intrinsic pathways. *Hypogymnia tubulosa* has been shown to have anti-proliferative and apoptotic effects in all extractions methods and our findings identified that both the percentage of the apoptotic cells and apoptotic protein expressions recorded an increase at lower treatment concentrations. Although *Lobaria pulmonaria* is known to have significant cytotoxic effects, we did not observe a decrease in cell proliferation. Indeed, proliferation marker proliferating cell nuclear antigen (PCNA) protein expression levels have shown an increase in all extracts, while *Usnea florida exhibited* apoptosis induction and slight proliferation reduction in extract treatments with lower concentrations. We tested 18 extracts of six lichen species during our study. Of these, *Evernia divaricata* and *Hypogymnia tubulosa* demonstrated significant apoptotic activity on prostate cancer cells including at low concentrations, which implies that it is worth pursuing the biologically active lead compounds of these extracts on prostate cancer *in vitro*. Further corroboratory studies are needed to validate the relative potential of these extracts as anti-metastatic and anti-tumorigenic agents.

## Introduction

Lichens grouped under the Fungi Kingdom are autotrophic mutualistic associations formed by heterotrophic fungi (mycobiont) and photosynthetic green algae and/or cyanobacteria (photobionts) [1]. Lichen extracts have been used in the production of perfumes, dyes, and traditional remedies for centuries [2–5], and the utilization of various lichen species in therapeutic applications are extensively documented in the ancient medical literature [5–7]. Therapeutic effects of lichens originate from the production of many chemical compounds, known as secondary metabolites or lichen acids [8, 9], as a result of the synergistic interaction of fungi and algae in lichens [10, 11]. Secondary metabolites of lichens, which contain aliphatic, cycloaliphatic, aromatic, and terpenic compounds, possess significant biological and pharmacological characteristics that carry antioxidant, pro-apoptotic, anti-proliferative and cytotoxic, anti-inflammatory, antiviral, antibacterial, analgesic, and antipyretic abilities [12]. Preclinical researches conducted in several studies have shown that the antineoplastic activities of these compounds have inhibitory effects against cancer cells [13–16]. For example, Yang Y. *et al*. suggested usnic acid, an active secondary metabolite found in *Usnea florida*, might have potential role in the inhibition of lung cancer cell metastasis [14]. Another study showed that physciosporin, a potent secondary metabolite found in several lichens species belonging to the genus Pseudocyphellaria, has anticancer activity by suppressing the growth and motility of colorectal cancer cells via novel mechanisms [16]. These anti-proliferative, antitumor and anticancer effects are derived from the synergistic and/or antagonistic characteristics of compounds in lichens that individually contribute to the overall combined effect [17–21]. Lichens act as apoptosis activators in cancer cells [15] by affecting the gene expressions of apoptotic proteins such as caspases, p53, p38, or anti-/pro-apoptotic proteins of the Bcl-2 family [21].

Numerous investigations were commenced in recent years studying the anticancer effects of natural products derived from herbs, fungi or marine organisms to identify alternative treatment methods to overcome the problems encountered in cancer treatment. Cancer is one of the leading causes of morbidity and mortality accounting for almost one out of every six deaths in the world recording approximately 8.8 million deaths in 2015. The number of new cases is expected to rise by about 70% over the next two decades. Prostate cancer (PCa) is the second most commonly observed cancer in the world ranking the sixth place among cancer-induced deaths in men [22].

Surgical intervention, radiotherapy, hormonal therapy, and chemotherapy are the conventional treatment options utilized in the rehabilitation of prostate cancer known to have various limitations including their side effects and long treatment duration. Therefore, further studies are needed to develop effective alternative treatments with moderate to no side effects that could complement these approaches. Anticarcinogenic effects of various lichen species have been widely studied in the literature. In this study, we aimed to explore the anti-proliferative and apoptotic properties of acetone, ethanol, and methanol extracts of *Bryoria capillaris (Ach*.*) Brodo & D*. *Hawksw*, *Cladonia fimbriata (L*.*) Fr*., *Evernia divaricata (L*.*) Ach*., *Hypogymnia tubulosa (Schaer*.*) Hav*., *Lobaria pulmonaria (L*.*) Hoffm*., and *Usnea florida (L*.*) Weber ex Wigg*. in PC3 prostate cancer cells, which are considered an aggressive form or advanced (castration-independent) stage of prostate adenocarcinoma.

## Materials and methods

### Collection and identification of lichen specimens

Six species of lichens; *Bryoria capillaris* (BC), *Cladonia fimbriata* (CF), *Evernia divaricata* (ED), *Hypogymnia tubulosa* (HT), *Lobaria pulmonaria* (LP), and *Usnea florida* (UF) were collected (Field permit number: 72784983–488.04–89586 Republic of Turkey Ministery, Agriculture and Forestry, (TAGEM), cleaned from foreign materials, and dried in room temperature. The lichen samples were investigated under Nikon SMZ445 stereomicroscope and identified according to the keys of references [23, 24].

### Preparation of extracts

Each species was pulverized, and 10 gr of powdered lichen thalli was extracted with 200 ml ethanol, methanol, and acetone separately by using the Soxhlet apparatus. Extracts were filtered and concentrated in a rotary vacuum evaporator at 40⁰C. Following the storage of dry extracts at 4°C, they were dissolved in 5% dimethyl sulphoxide (DMSO) for further experiments.

### Cell culture

PC-3 human androgen-independent cells, grown in RPMI 1640 (Gibco, Thermo Fisher Scientific, NY, USA) were supplemented with 10% fetal bovine serum (FBS) (Gibco), 1% penicillin-streptomycin, and 0.01% primocin (Invivogen, CA, USA). Cultures were incubated at 37°C in a 5% CO2 atmosphere and 95% relative humidity.

Before treatments, 5x10^3^ cells were seeded into 96-well plates for 24hr, 48hr, and 72 hr. After 24 hr cells were washed with 1X (PBS) and treated with 200 μL medium containing one of seven different concentrations of lichen extracts. The final concentrations of the extracts in the cell cultures were 100 μg/mL, 50 μg/mL, 25 μg/mL, 12.5 μg/mL, 6.25 μg/mL, 3.125 μg/mL, and 1.56 μg/mL. These concentrations were obtained by diluting the extracts in DMSO (1 mg dried extract dissolved in 1 mL DMSO) and accepted as 100X stock with a final concentration of 1mg/mL. Three non-cytotoxic concentrations were chosen based on the (4,5-dimethylthiazol-2-yl)-2,5 diphenyltetrazolium bromide (MTT) and Lactate dehydrogenase (LDH) assay analyzes. All experiments were performed as triplicates. Inhibitory Concentration (IC50) values, varied between extracts of each lichen species, were calculated by using MTT assay results. Doses used in further experiments were determined based on the comparison between MTT and LDH assays. During the analysis of the viability/cytotoxicity values; the background control (the group that contains only MTT/LDH solution with no cells) was subtracted from samples in the first place, and the calculated average of the blank group (the group that only includes cells without extract treatment) was accepted as healthy cells with 100% viability.

### MTT assay

Cells treated with DMSO or indicated concentrations of lichen extracts for three-time intervals were incubated with the diluted MTT solution (0.2 ml/well) at 37°C and 5% CO_2_ for four hours. DMSO was added (0.1 ml/well) to solubilize the formazan crystals. The plates were gently agitated and incubated at 37°C for another 10 minutes. The absorbance of the supernatant was measured at 540 nm. The percentage of viable cells was obtained using the following formula:

$$\frac{\mathrm{Absorbance}\;\mathrm{of}\;\mathrm{treated}\;\mathrm{cells}\;X\;100}{\mathrm{Absorbance}\;\mathrm{of}\;\mathrm{control}\;\mathrm{cells}}$$

### LDH assay

Extracellular LDH in the media can be quantified in which LDH catalyzes the conversion of lactate to pyruvate via Nicotinamide adenine dinucleotide (NAD+) reduction to NADH. Diaphorase then uses NADH to reduce a tetrazolium salt to a red formazan product that can be measured at 490 nm. The level of formazan formation is directly proportional to the amount of LDH released into the medium. After treatment of cells with extracts, the 50 μl medium was separated from each group into a new 96-well plate. The LDH reaction was performed using a Pierce LDH Cytotoxicity Assay Kit (Thermo Fisher Scientific, NY, USA) following the manufacturer's instructions. The percentage of cytotoxicity was obtained using the following formula:

$$\frac{\mathrm{Experimental}\;\mathrm{value}‐\mathrm{Effector}\;\mathrm{cells}\;\mathrm{spontaneous}\;\mathrm{control}‐\mathrm{Target}\;\mathrm{cells}\;\mathrm{spontaneous}\;\mathrm{control}}{\mathrm{Target}\;\mathrm{cell}\;\mathrm{maximum}\;\mathrm{control}‐\mathrm{Target}\;\mathrm{cells}\;\mathrm{spontaneous}\;\mathrm{control}}$$

### Apoptosis analysis

Apoptosis analyses were performed with a Muse Cell Analyzer (Merck Millipore, Germany) using the Muse Annexin V & Dead Cell Assay kit (Merck Millipore, Germany). In this assay, a premixed Annexin V in combination with a dead cell marker, 7-amino actinomycin (7-AAD) is used. Prior to assaying, cells were treated with the extracts at specified three non-cytotoxic concentrations at different time intervals. Cells were resuspended in PBS with 1% FBS, mixed with the Muse Annexin V and Dead Cell reagent. Samples were incubated for 20 minutes at room temperature in the dark. Apoptotic cell ratios were analyzed by flow cytometry using Muse Cell Analyzer system and gating was adjusted according to the untreated sample. Results were presented as the percentage of cells that were viable (Ann-V− 7-AAD−), early apoptotic (Ann-V+ 7-AAD−), late apoptotic (Ann-V+ 7-AAD−), or dead (Ann-V− 7-AAD+) [25].

### Western blotting

Cells were cultured with different concentrations of lichen extracts at three-time intervals, washed with PBS and to obtain total protein extracts 3x10^6^ cells were lysed in 100 μl complete radioimmunoprecipitation assay (RIPA) lysis buffer (containing 5μl phenylmethylsulfonyl fluoride, 5μl sodium orthovanadate solution, and 5μl protease inhibitor cocktail) (Santa Cruz Texas, USA). Cell suspension was frozen overnight at -80°C and supernatant was collected after centrifuging for 15 minutes at 13000 g. Total protein concentrations were measured using a Coomassie (Bradford) Protein Assay Kit (Thermo Fisher Scientific MA, USA). 50 μg sample of total protein extract was prepared with 4X laemmli sample buffer and separated on 4–20% precast polyacrylamide gel (Biorad, USA). Proteins transferred to 0.2 μm polyvinylidene difluoride (PVDF) membranes (Bio-Rad, USA) in 1X transfer buffer at 2.5 A for 10 minutes with the Trans-Blot Turbo Transfer System (Bio-Rad, USA). After blocking overnight at +4°C with TBST (Tris Buffered Saline with 0.02% Tween-20) containing 5% nonfat milk, membranes were probed with primary antibodies against β-actin (1:1000 dilution, Cell Signaling, cat no: 4967, MA, USA), poly (ADP-ribose) polymerase (PARP) (1:1000, Cell Signaling, cat no:9542, MA, USA), Proliferating cell nuclear antigen (PCNA) (1:2000, Cell Signaling, cat no:2586, MA, USA), Caspase-3 (1:1000, Cell Signaling, cat no:9662, MA, USA), Cleaved caspase 9 (1:1000, Cell Signaling, cat no.9505 MA, USA), Cleaved caspase 8 (1:1000, Cell Signaling, cat no:9496, MA, USA). Secondary antibodies were anti-rabbit IgG and anti-mouse IgG HRP-linked antibodies (both were used in 1:3000 dilution, Cell Signaling, MA, USA). The blots were developed using the enhanced chemiluminescence (ECL) detection kit (Advansta, CA, USA). Band densities were measured using Image-J and normalised against that of β-actin in each sample.

### Statistics

Statistical testing for multiple comparisons by the false discovery rate (FDR) for type-I error rate estimation is performed by the Benjamani, Krieger and Yekutieli methods [26]. The adjusted significance level was set as a nominal p-value<0.05 and FDR<0.05 were used to assess the significance. Statistical analyses were conducted using two-way Analysis of Variance (ANOVA) is applied with Dunnett's test and student t-test when necessary as nonparametric methods for the analysis of MTT, LDH, Flow cytometry, and Western blot data. *p < 0*.*05* was considered sufficient to reject the null hypothesis. All data are presented as the mean ± SD, with a significance level of *p ≤ 0*.*05* (*p < 0.05, **p < 0.*001*, ***p < 0.0001). All results were evaluated using the interface GraphPad Prism 7® software system (La Jolla, CA, USA).

## Results

### Collection of lichens

**Fig 1:**
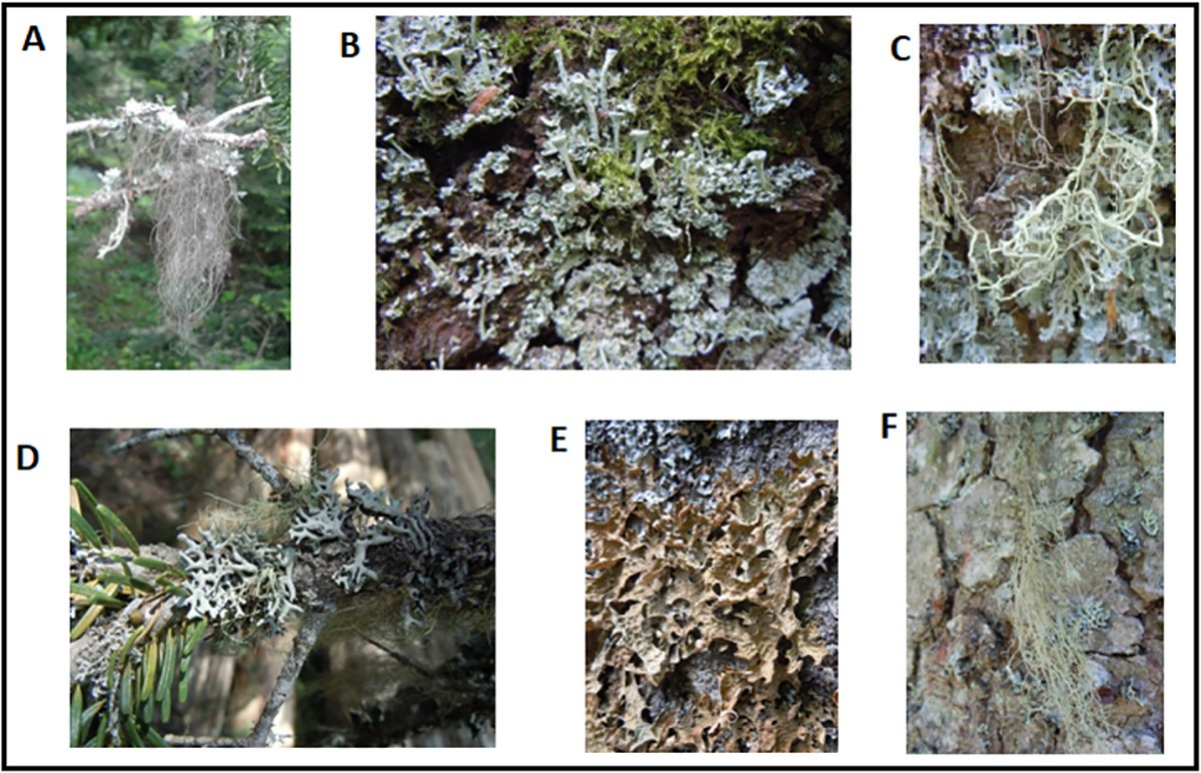
Field photos of lichen specimens A. *Bryoria capillaris* B. *Cladonia fimbriata* C. *Evernia divaricata* D. *Hypogymnia tubulosa* E. *Lobaria pulmonaria* F. *Usnea florida*.

Six species of lichens *Bryoria capillaris*, *Cladonia fimbriata*, *Evernia divaricata*, *Hypogymnia tubulosa*, *Lobaria pulmonaria* and, *Usnea florida* were collected, location and season of the collection are exhibited in Table 1. The field photos of the lichen species are illustrated in Fig 1 and the actual yields of the prepared dry extracts are displayed in Table 2.

**Table 1:** Lichen species, location (province) and collection date.

Species	Province	Collection date
*Bryoria capillaris*	Bolu Serif Yuksel Research Forest	2016- June
*Cladonia fimbriata*	Bolu Serif Yuksel Research Forest	2017-July
*Evernia divaricata*	Bolu Serif Yuksel Research Forest	2016- June
*Hypogymnia tubulosa*	Aladag / Bolu	2017-July
*Lobaria pulmonaria*	Kazdagi / Canakkale	2016- June
*Usnea florida*	Aladag / Bolu	2017-July

*Bryroria capillaris*, *Cladonia fimbricata*, and *Evernia divaricata* were collected from Bolu Serif Yuksel Research Forest; *Hypogymnia tubulosa*, and *Usnea florida* were collected from Aladag/Bolu and *Lobaria pulmonaria* was collected from Kazdagi/Canakkale in 2016 and 2017.

**Table 2:** The actual yields of the prepared dry extracts of lichens.

Species/ Solvent	*Bryoria capillaris*	*Cladonia fimbriata*	*Evernia divaricata*	*Hypogymnia tubulosa*	*Lobaria pulmonaria*	*Usnea florida*
**Acetone**	3.20	3.55	8.50	8.66	25.20	3.99
**Ethanol**	6.92	23.65	13.76	12.32	8.60	7.48
**Methanol**	9.30	11.67	8.50	15.96	16.60	9.41

The yields of dry extracts of lichen species vary, based on different extraction solvents. Values are shown as milligram (mg).

### Cell viability and cytotoxicity assays

Comparative viability/cytotoxicity experiments were performed with MTT and LDH assays. The yields of different bioactive molecules and the effect of each extract, which may be either acquired or lost depends on the extraction method and the type of solvent used for extraction. Three different extraction methods; acetone (A), ethanol (E), and methanol (M) were utilized.

**Fig 2:**
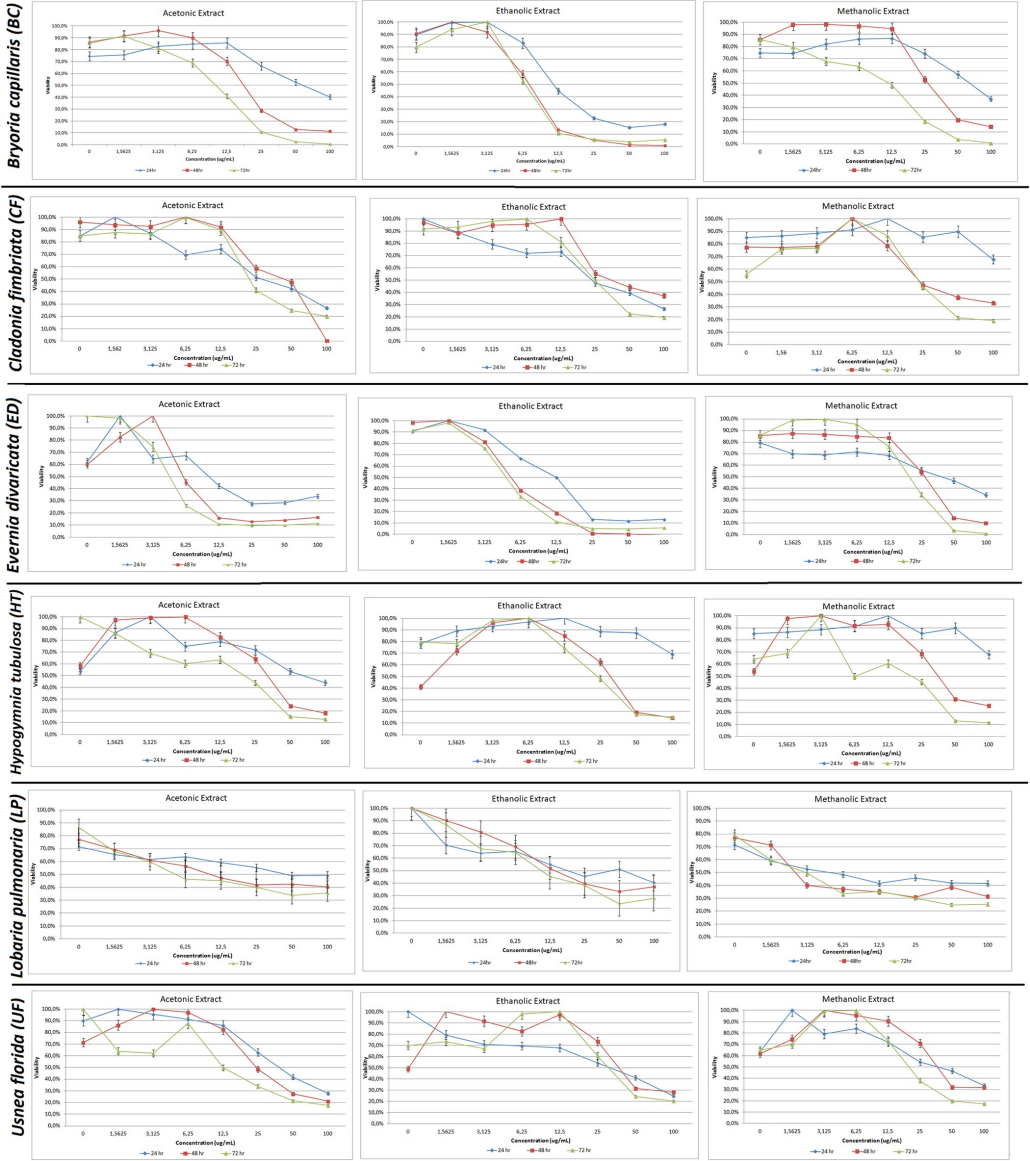
Cell viability by 3-(4,5-dimethylthiazol-2-yl)-2,5-diphenyltetrazolium bromide (MTT) assay. PC-3 cells were treated with acetone, ethanol and methanol extracts of *Bryoria capillaris*, *Cladonia fimbriata*, *Evernia divaricata*, *Hypogymnia tubulosa*, *Lobaria pulmonaria*, and *Usnea florida* at 24, 48, 72 hours.

**Fig 3:**
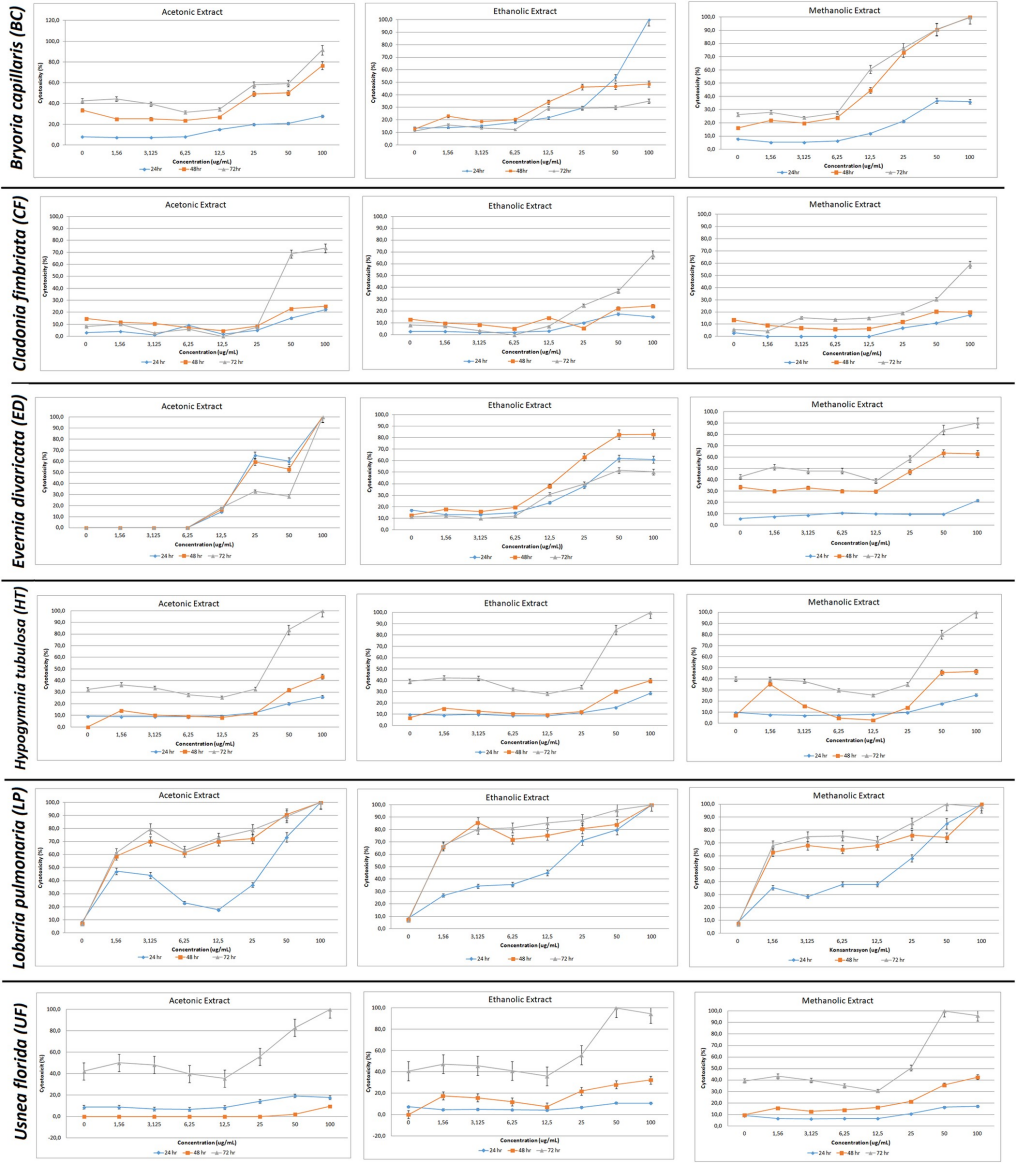
Cytotoxicity results by lactate dehydrogenase (LDH) assay. PC-3 cells were treated with acetone, ethanol and methanol extracts of *Bryoria capillaris*, *Cladonia fimbriata*, *Evernia divaricata*, *Hypogymnia tubulosa*, *Lobaria pulmonaria*, and *Usnea florida* cells at 24, 48, 72 hours.

The cell viability rates of the extract treatments at 24hr, 48hr, and 72hr intervals are shown in Fig 2. The cytotoxicity results of LDH assay and IC50 levels were evaluated (Fig 3 and Table 3).

**Table 3:** IC50 values of extracted lichen species.

Extract	IC50 (μg/ml)	Incubation Time (hr)
BC-A	11.37	72
BC-E	6.303	72
BC-M	14.74	72
CF-A	22.58	72
CF-E	21.59	72
CF-M	24.71	72
ED-A	8.849	24
ED-E	5.338	48
ED-M	20.54	72
HT-A	1.591	72
HT-E	23.19	72
HT-M	26.26	72
LP-A	0.0766	72
LP-E	7.416	72
LP-M	0.002154	72
UF-A	22.53	48
UF-E	30.89	48
UF-M	26.4	48

IC50 values of different lichen species extracts were evaluated based on non-cytotoxic concentrations and incubation times of each extract. BC:*Bryoria capillaris*, CF:*Cladonia fimbriata*, ED:*Evernia divaricata*, HT:*Hypgminia tubulosa*, LP:*Lobaria pulmonaria* and UF:*Usnea florida*

#### *Bryoria capillaris* (BC)

While cytotoxicity levels of different extraction methods in BC recorded an increase at all time intervals after 6.25μg/ml, viability levels began to decrease in a dose and time-dependent manner particularly during the use of methanol extraction (Figs 2 and 3). The earliest cytotoxic response (at 24 hr) that starts proliferation at 6.25μg/ml concentration is achieved by ethanolic extraction. All extracts exhibit decreased cell viability around 15%-0 rate after 48 hr.

#### *Cladonia fimbriata* (CF)

Cytotoxicity started to increase after 12.5 μg/ml in each extract type where 100 μg/ml is the highest reached level. While the cytotoxicity patterns of ethanol and methanol were similar, a different pattern was observed in all acetone concentrations, which recorded a sharp upsurge after 25 μg/ml. Acetonic extraction recorded 100% decreased viability at 48 hr, while the remaining extraction methods recorded lower viability ratios with 70% being the highest recorded (Figs 2 and 3).

#### *Evernia divaricata* (ED)

The cell viability reached the peak level at the lowest concentrations (1.56 μg/ml and 3.125 μg/ml) at all time intervals using acetone extraction. For ethanol and methanol extractions, viability decreased sharply after 1.56 μg/ml and 6.25 μg/ml respectively. The same pattern in the opposite direction was recorded for cytotoxicity (Figs 2 and 3).

#### *Hypogymnia tubulosa* (HT)

A decrease in cell viability was observed at 12.5 μg/ml in acetone extraction (Figs 2 and 3). Cells at 48 hr and 72 hr time intervals demonstrated a similar pattern, while cell viability differentiated at 24 hr. 12.5 μg/ml is the concentration where an increase in cytotoxicity levels were observed at all time intervals.

#### *Lobaria pulmonaria* (LP)

LP demonstrated the highest level of live cells among six lichen species utilizing all three extraction methods. The proportion of live cells at the highest concentration was around 30% as demonstrated by all extraction methods even at 72 hr time point. This result was also confirmed by Annexin V assay (Figs 2 and 3).

#### *Usnea florida* (UF)

The cell viability recorded a decrease, while the cytotoxicity levels recorded an increase at 12.5 μg/ml concentration for this lichen species utilizing all the extraction methods (Figs 2 and 3).

### Determination of apoptosis by flow cytometry and Western blot analysis

To confirm the experiment results above, more cell death specific parameters were investigated. Assessment of apoptotic behavior was observed with the treatment of each extract by using Annexin V/7’AAD. The decrease in cell confluency in PC-3 cells was accompanied by morphological changes, including the loss of adherence and the rounding of the cell shape. Treatment of each extract at their non-cytotoxic doses was evaluated and apoptotic rates were examined with Annexin V/7'AAD.

PC-3 cells efficiently sustained apoptosis upon different extract treatments.

#### *Bryoria capillaris* (BC)

**Fig 4:**
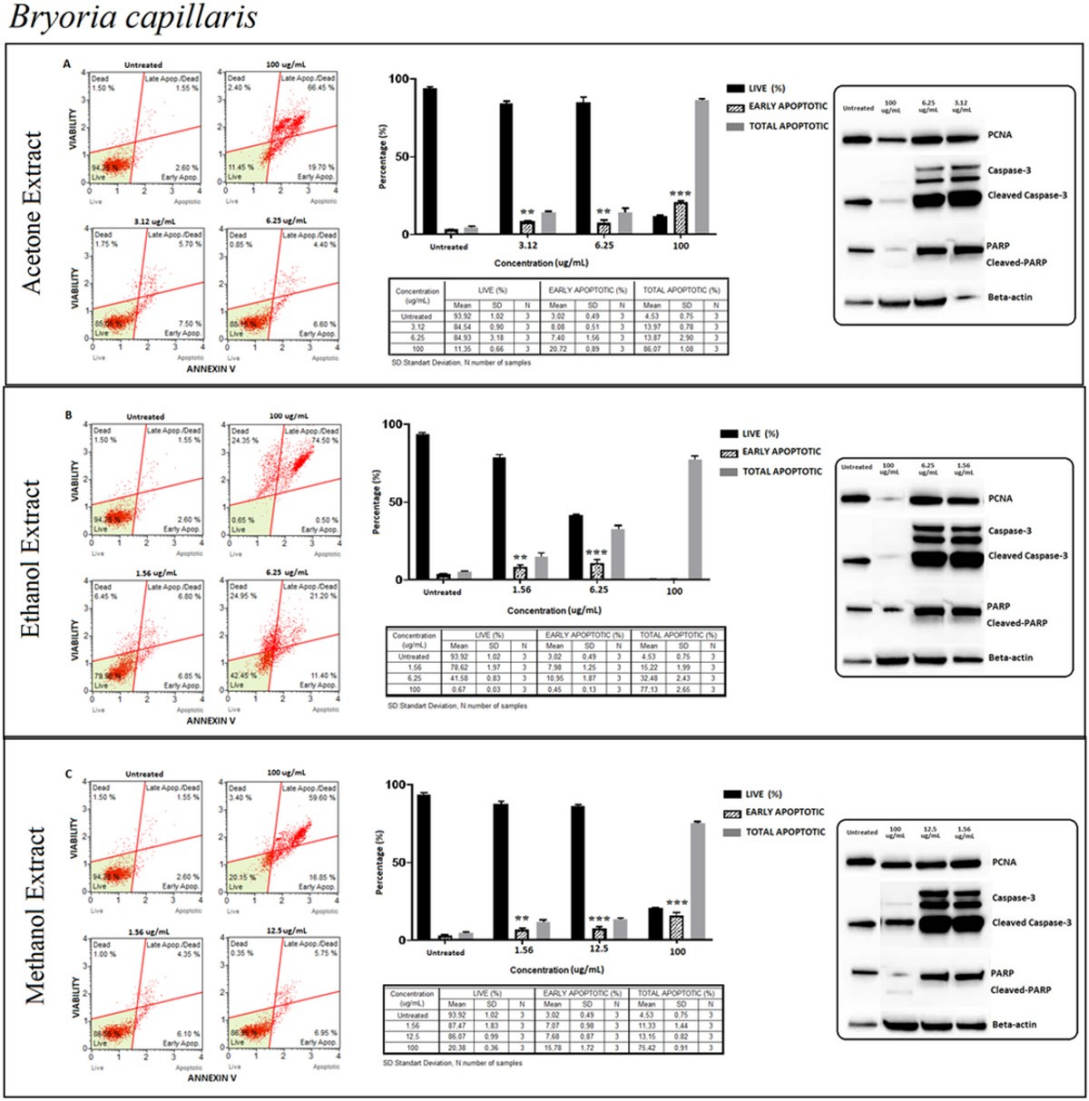
*Bryoria capillaris* (BC); Annexin V/7’AAD staining flow cytometry results (scatterplots), statistical analysis (bar graphs), ±SD values (tables) and protein expression levels of PCNA, caspase-3 and PARP by western blot (right panel) on PC-3 cells with acetone (A), ethanol (B) and methanol (C) extraction methods respectively. Basically, early apoptosis on PC3 cells is shown via Annexin V/7’AAD staining by flow cytometry. The gating was adjusted according to the untreated sample and the representative scatterplots were presented as the percentage of cells that were viable (Ann-V^−^ and 7-AAD^−^), early apoptotic (Ann-V^+^ and 7-AAD^−^), late apoptotic (Ann-V^+^ and 7-AAD^−^), and dead (Ann-V^−^ and 7-AAD^+^). Scatterplots are shown for untreated, 100 μg/mL, 6.25 μg/mL and 3.12 μg/mL at 72 hours treatment with acetone extract (A) and for ethanol extract treatment, groups are untreated, 100 μg/mL, 6.25 μg/mL and 1.56 μg/mL at 72 hours treatment (B) and for methanol extract treatment, groups are untreated, 100 μg/mL, 12.5 μg/mL and 1.56 μg/mL at 72 hours treatment (C) respectively. The 100 μg/mL concentration is determined as toxic dose for all treatment groups. Results were analyzed and presented as the percentage of three independent experiments of live, early apoptotic and total apoptotic after treatment (bar graphs). Flow cytometry was shown to induce apoptotic cell death by mainly early apoptosis. The significance of only early apoptosis for each concentration indicated with an asterisk and the *p* value was only presented when the significance level of early apoptosis rate between 0.05 and 0.01. ±SD changes between apoptosis assays were indicated as the mean value and SD changes per concentration and per group of live-early apoptotic-total apoptotic separately (table). Western blot results of proliferating cell marker PCNA and apoptosis-related proteins (right panel) were presented. Dose-dependent PCNA, cleavage of caspase-3 and, PARP respectively. Apoptosis rate seems increased significantly by treatment with specified concentrations by flow cytometry however western blot data showed otherwise, particularly for cleaved-PARP. In addition, the proliferation (PCNA) status remained stable. *p < 0.*05*, **p < 0.*001*, ***p < 0.*0001* (all p-values were obtained by two-way ANOVA following Dunnet's multiple comparison test).

While apoptosis was detected at all indicated doses at a minimum level, late apoptosis even early necrosis was observed at 100 μg/ml. It was a remarkable observation that the strength of caspase activation depended on both the type and amount of the extract. Activation of caspase-3 peaked upon stimulation with the lower concentrations of acetone and ethanolic extracts (Fig 4).

#### *Cladonia fimbriata* (CF)

**Fig 5:**
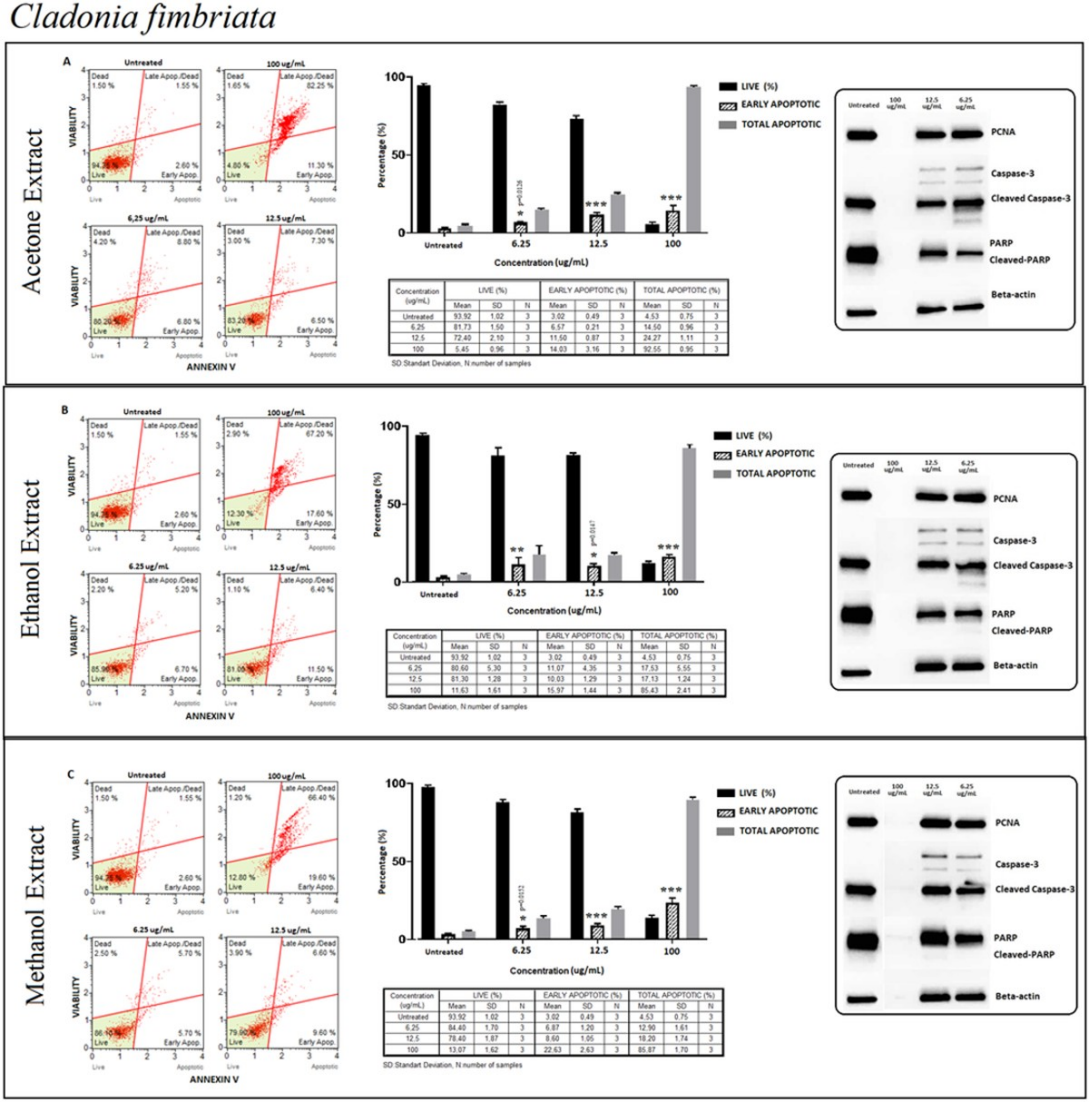
*Cladonia fimbriata* (CF); Annexin V/7’AAD staining flow cytometry results (scatterplots), statistical analysis (bar graphs), ±SD changes between experiments (tables) and protein levels of PCNA, caspase-3 and PARP by western blot (right panel) on PC-3 cells with (results compiled in A), ethanol (results compiled in B) and methanol (results compiled in C) respectively. Annexin V/7’AAD staining by flow cytometry of CF on PC-3 cells was shown to induce apoptotic cell death by mainly early apoptosis. The gating was adjusted according to the untreated sample and the representative scatterplots were presented as the percentage of cells that were (Ann-V^−^ and 7-AAD^−^), early apoptotic (Ann-V^+^ and 7-AAD^−^), late apoptotic (Ann-V^+^ and 7-AAD^−^), and dead (Ann-V^−^ and 7-AAD^+^). Scatterplots are shown for untreated, 100 μg/mL, 12.5 μg/mL and 6.25 μg/mL after 72 hours treatment with acetone (A), ethanol (B) and methanol extracts (C) respectively. The 100 μg/mL concentration is determined as toxic dose for all extract groups. Results were analyzed and presented as the percentage of three independent experiments of live, early apoptotic and total apoptotic after treatment (bar graphs). Flow cytometry was shown to induce apoptotic cell death by mainly early apoptosis. The significance of only early apoptosis for each concentration indicated with an asterisk and the *p* value was only presented when the significance level of early apoptosis rate between 0.05 and 0.01. ±SD changes between apoptosis assays were indicated as the mean value and SD changes per concentration and per group of live-early apoptotic-total apoptotic separately (table). Western blot results of proliferating cell marker PCNA and apoptosis-related proteins (right panel) were presented. Dose-dependent PCNA, cleavage of caspase-3 and, PARP respectively. Apoptosis rate seems increased significantly by treatment with specified concentrations by flow cytometry when compared with untreated group. However western blot data showed otherwise, particularly for cleaved caspase-3 and cleaved-PARP. In addition, proliferation status (PCNA) remained stable for the specified concentrations when compared with untreated group. *p < 0.*05*, **p < 0.*001*, ***p < 0.*0001* (all *p*-values were obtained by two-way ANOVA following Dunnet's multiple comparison test).

Similar patterns confirming cytotoxicity levels and cell viability were determined for apoptosis detection. By Annexin V assay, it was presented that dead cells that were likely to be the necrotic cells reached the highest level at 25 μg/ml in acetone extraction. Having found no band intensity at 25μg/ml activated caspase-3 was detected mostly at the lower concentrations. Western blot and Annexin V assay results were compiled in (Fig 5).

#### *Evernia divaricata* (ED)

**Fig 6:**
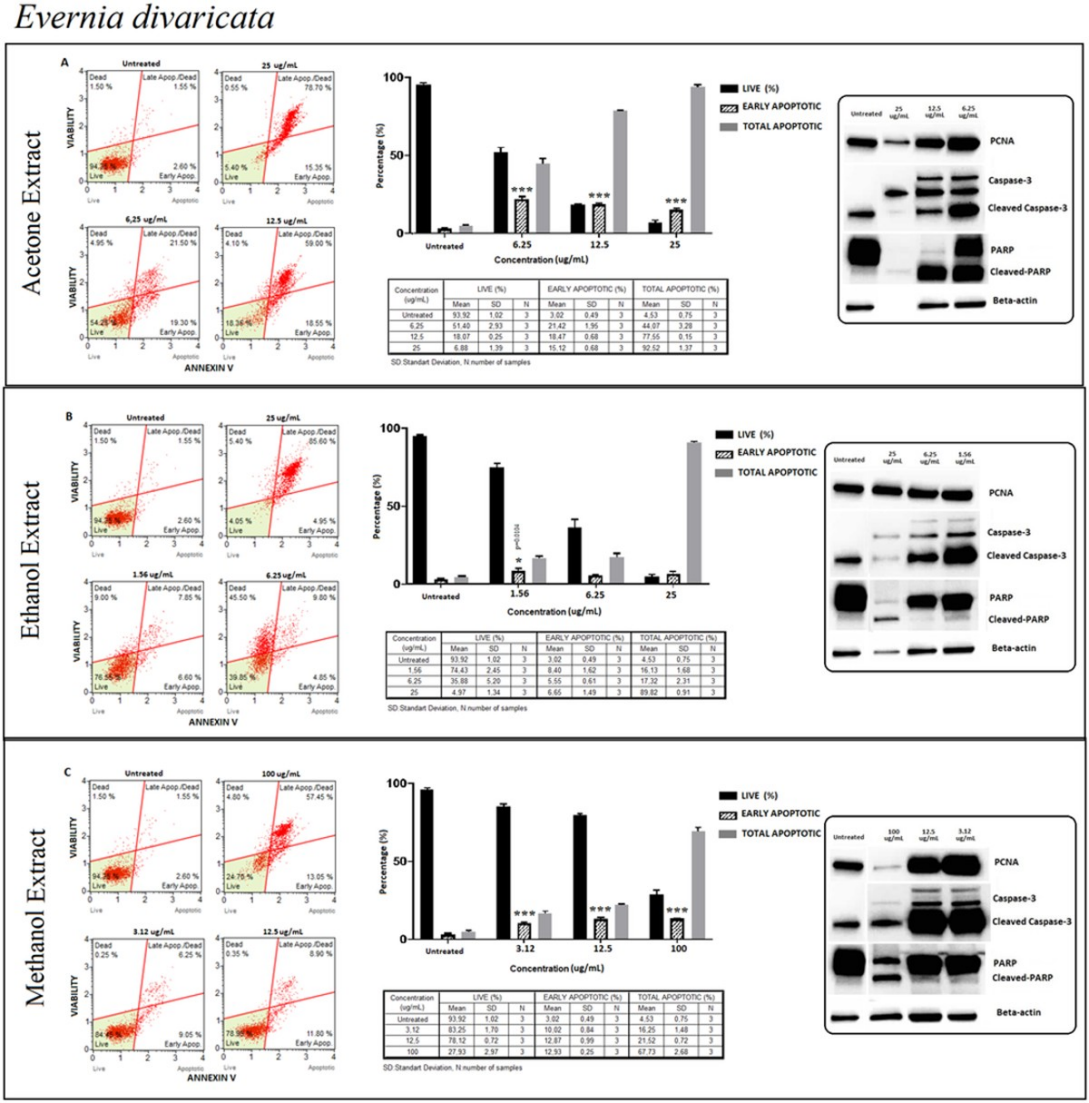
*Evernia divaricata* (ED); Annexin V/7’AAD staining flow cytometry results (scatterplots), statistical analysis (bar graphs), ±SD changes between experiments (tables) and protein levels of PCNA, caspase-3 and PARP by western blot (right panel) on PC-3 cells with three different extraction of acetone (results compiled in A), ethanol (results compiled in B) and methanol (results compiled in C) respectively. Annexin V/7’AAD staining by flow cytometry of ED on PC-3 cells was shown to induce apoptotic cell death by mainly early apoptosis. The gating was adjusted according to the untreated sample and the representative scatterplots were presented as the percentage of cells that were viable (Ann-V^−^ and 7-AAD^−^), early apoptotic (Ann-V^+^ and 7-AAD^−^), late apoptotic (Ann-V^+^ and 7-AAD^−^), and dead (Ann-V^−^ and 7-AAD^+^). Scatterplots are shown for untreated, 25 μg/mL, 12.5 μg/mL and 6.25 μg/mL after 24 hours treatment with acetone extract (A) and for ethanol extract treatment; groups are untreated, 25 μg/mL, 6.25 μg/mL and 1.56 μg/mL after 48 hours treatment (B) and for methanol extract treatment; groups are untreated, 100 μg/mL, 12.5 μg/mL and 3.12 μg/mL after 72 hours treatment (C) respectively. The 25 μg/mL concentration is determined as toxic dose for acetone and ethanol extracts groups and the 100 μg/mL concentration is determined as toxic dose for methanol extract group. Results were analyzed and presented as the percentage of three independent experiments of live, early apoptotic and total apoptotic after treatment (bar graphs). Flow cytometry was shown to induce apoptotic cell death by mainly early apoptosis (except ethanol extract). The significance of only early apoptosis for each concentration indicated with an asterisk and the *p* value was only presented when the significance level of early apoptosis rate between 0.05 and 0.01. ±SD changes between apoptosis assays were indicated as the mean value and SD changes per concentration and per group of live-early apoptotic-total apoptotic separately (table). Western blot results of proliferating cell marker PCNA and apoptosis-related proteins (right panel) were presented. Dose-dependent PCNA, cleavage of caspase-3 and, PARP respectively. Particularly, acetone extract group showed significant increase with 24 hours of treatment for apoptosis by flow cytometry and western blot respectively, even with higher proliferation rate of PCNA. Ethanol and methanol extract groups showed increased apoptosis rate (only by flow cytometry) with specified concentrations however even marked increase for cleaved caspase-3 rate, cleaved-PARP did not showed significant rate, as presented in the western blot (right panel) when compared with untreated group. *p < 0.*05*, **p < 0.*001*, ***p < 0.*0001* (all *p*-values were obtained by two-way ANOVA following Dunnet's multiple comparison test).

**Fig 7:**
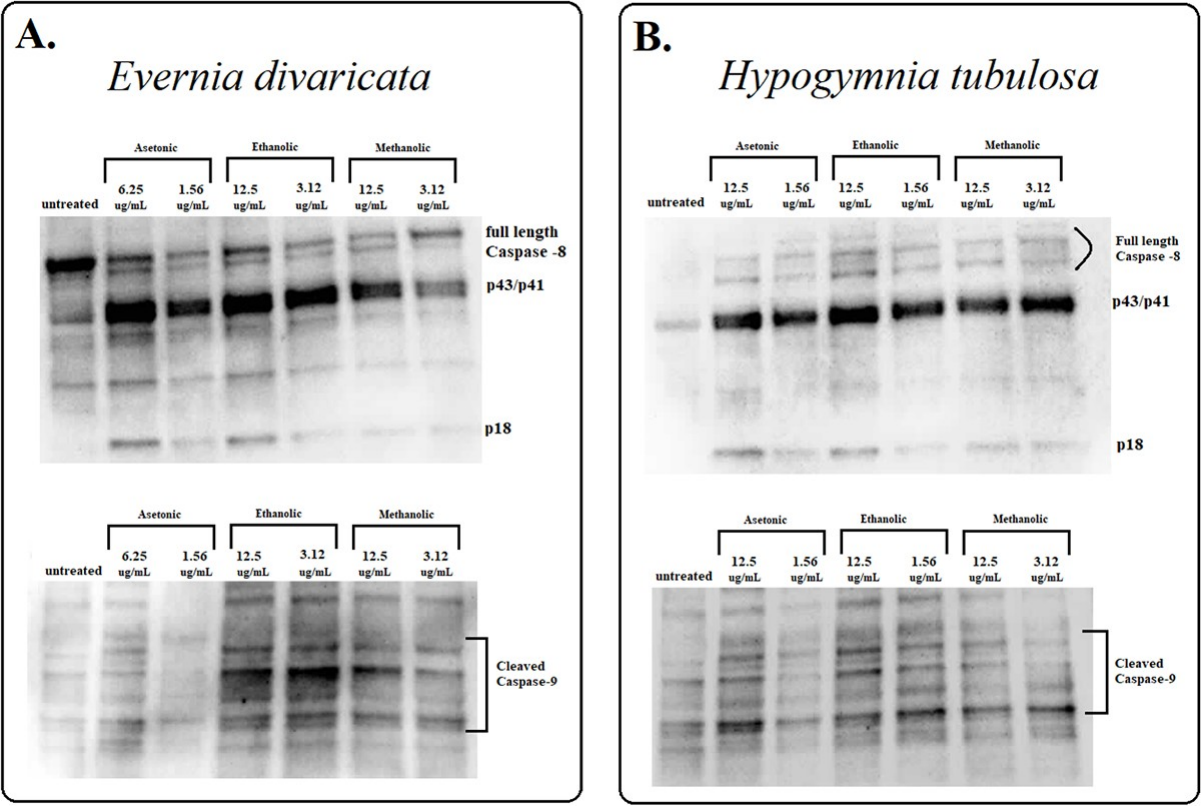
Determination of caspase-8 and caspase-9 protein levels from *Evernia divaricata* and *Hypogymnia tubulosa* with acetone, ethanol and methanol extracts on PC3 cells. **A.** Caspase-8 protein expression of *Evernia divaricata* with acetone, ethanol and, methanol extraction at 24, 48, 72 hours of treatment respectively (upper side). Cleaved caspase-9 protein expression of *Evernia divaricata* with acetone, ethanol and, methanol extraction at 24, 48, 72 hours of treatment respectively (lower side) **B.** Caspase-8 protein expression of *Hypogymnia tubulosa* with acetone, ethanol and, methanol extraction at 72 hours of treatment respectively (upper side). Cleaved caspase-9 protein expression of *Hypogymnia tubulosa* with acetone, ethanol and, methanol extraction at 72 hours of treatment respectively (lower side).

In all the extracts except ethanol, a similar pattern of a dose-dependent manner induction was recorded for apoptosis detection (Fig 6). In all three extractions, the ratio of early apoptotic cells remained the same while the ratio of live cells decreased indicating that this event might have happened as a result of apoptosis but also because of other accompanying cell death types. Apoptotic detection was also confirmed by cleaved caspase-3 and cleaved-PARP where increased apoptosis levels were recorded mostly at lower doses (Fig 6). Among the upstream regulators of caspase-3 and PARP activation, ED extracts were further evaluated for the protein expression levels of caspase-8, and caspase-9 whether apoptosis was through intrinsic or extrinsic pathways (Fig 7). ED induced apoptotic cell death via extrinsic pathways were observed in all extraction methods, confirmed by increased levels of a cleaved caspase 8 (p43/p41).

#### *Hypogymnia tubulosa* (HT)

**Fig 8:**
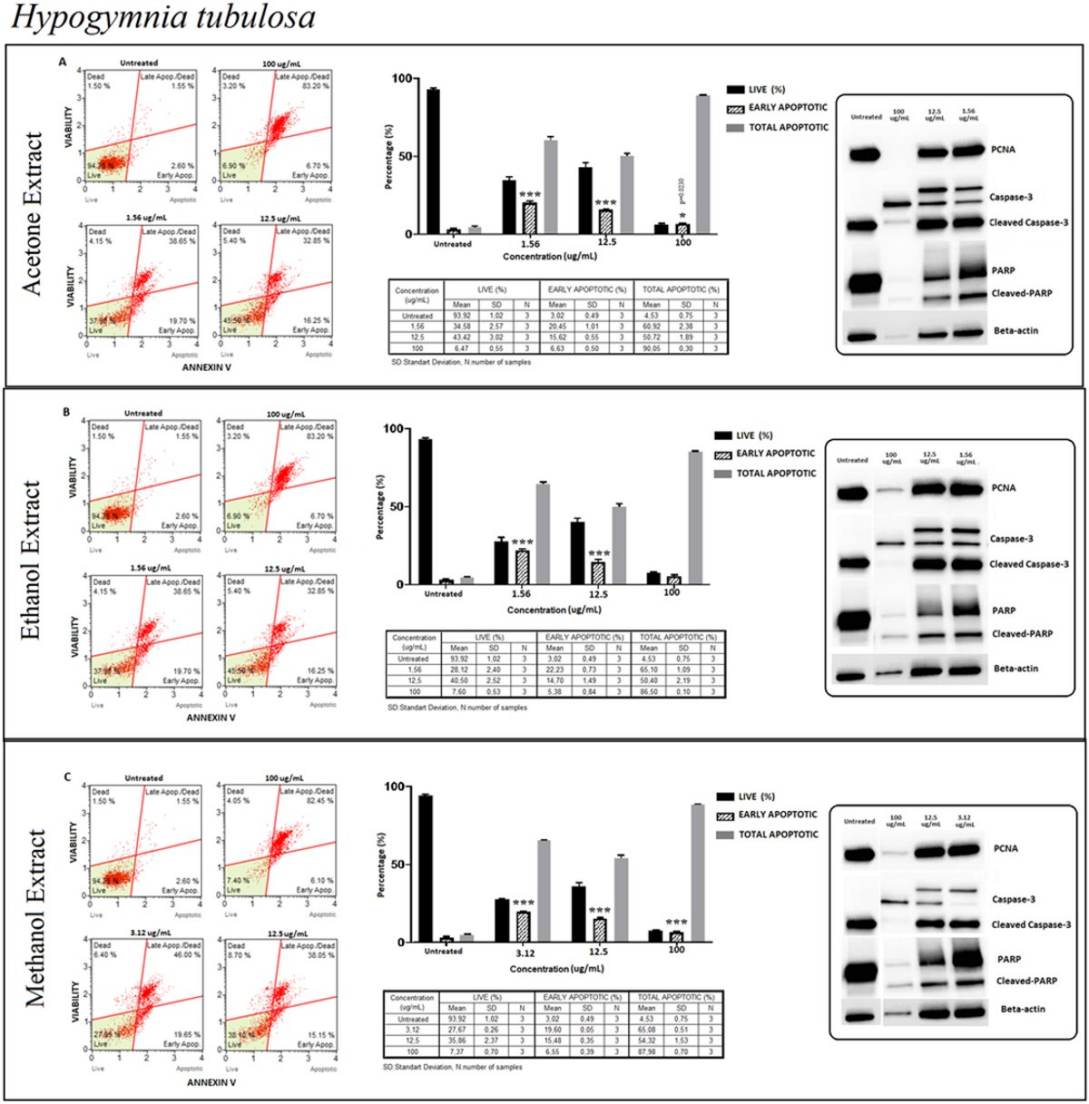
*Hypogymnia tubulosa* (HT); Annexin V/7’AAD staining flow cytometry results (scatterplots), statistical analysis (bar graphs), ±SD changes between experiments (tables) and protein levels of PCNA, caspase-3 and PARP by western blot (right panel) on PC-3 cells with three different extraction of acetone (results compiled in A), ethanol (results compiled in B) and methanol (results compiled in C) respectively. Annexin V/7’AAD staining by flow cytometry of HT on PC-3 cells was shown to induce apoptotic cell death by mainly early apoptosis. The gating was adjusted according to the untreated sample and the representative scatterplots were presented as the percentage of cells that were viable (Ann-V^−^ and 7-AAD^−^), early apoptotic (Ann-V^+^ and 7-AAD^−^), late apoptotic (Ann-V^+^ and 7-AAD^−^), and dead (Ann-V^−^ and 7-AAD^+^). Scatterplots are shown for untreated, 100 μg/mL, 12.5 μg/mL and 1.56 μg/mL after 72 hours treatment with acetone extract (A) and ethanol extract (B) as well. For methanol extract treatment, groups are untreated, 100 μg/mL, 12.5 μg/mL and 3.12 μg/mL after 72 hours treatment (C). The 100 μg/mL concentration is determined as toxic dose for all extract group. Results were analyzed and presented as percentage of three independent experiments of live, early apoptotic and total apoptotic after treatment (bar graphs). Flow cytometry was shown to induce apoptotic cell death by mainly early apoptosis. The significance of only early apoptosis for each concentration indicated with an asterisk and the *p* value was only presented when the significance level of early apoptosis rate is between 0.05 and 0.01. ±SD changes between apoptosis assays were indicated as the mean value and SD changes per concentration and per group of live-early apoptotic-total apoptotic separately (table). Western blot results of proliferating cell marker PCNA and apoptosis-related proteins (right panel) were presented. Dose-dependent PCNA, cleavage of caspase-3 and PARP respectively. Remarkably, apoptosis rate significantly increased with the specified concentrations for all extraction and the presented results showed positive correlation with flow cytometry and western blot. In addition, the proliferation did not change significantly when compared with untreated group. *p < 0.*05*, **p < 0.*001*, ***p < 0.*0001* (all *p*-values were obtained by two-way ANOVA following Dunnet's multiple comparison test).

The percentage of live cells recorded a decrease of around 60% at the lowest concentrations and a significant increase at 12.5 μg/ml in all extraction methods. At this stage, the percentage of early apoptotic cells was 30% as the percentage of late apoptotic cells increased by more than 50% (Fig 8). Increased apoptosis level was recorded with verified cleaved caspase-3 and cleaved-PARP detections (Fig 8). To confirm the apoptotic pathways of HT extract, the extrinsic and intrinsic pathways were supported by processing procaspase 8 to its cleavage segments p43/p41 and p18 (Fig 7).

#### *Lobaria pulmonaria* (LP)

**Fig 9:**
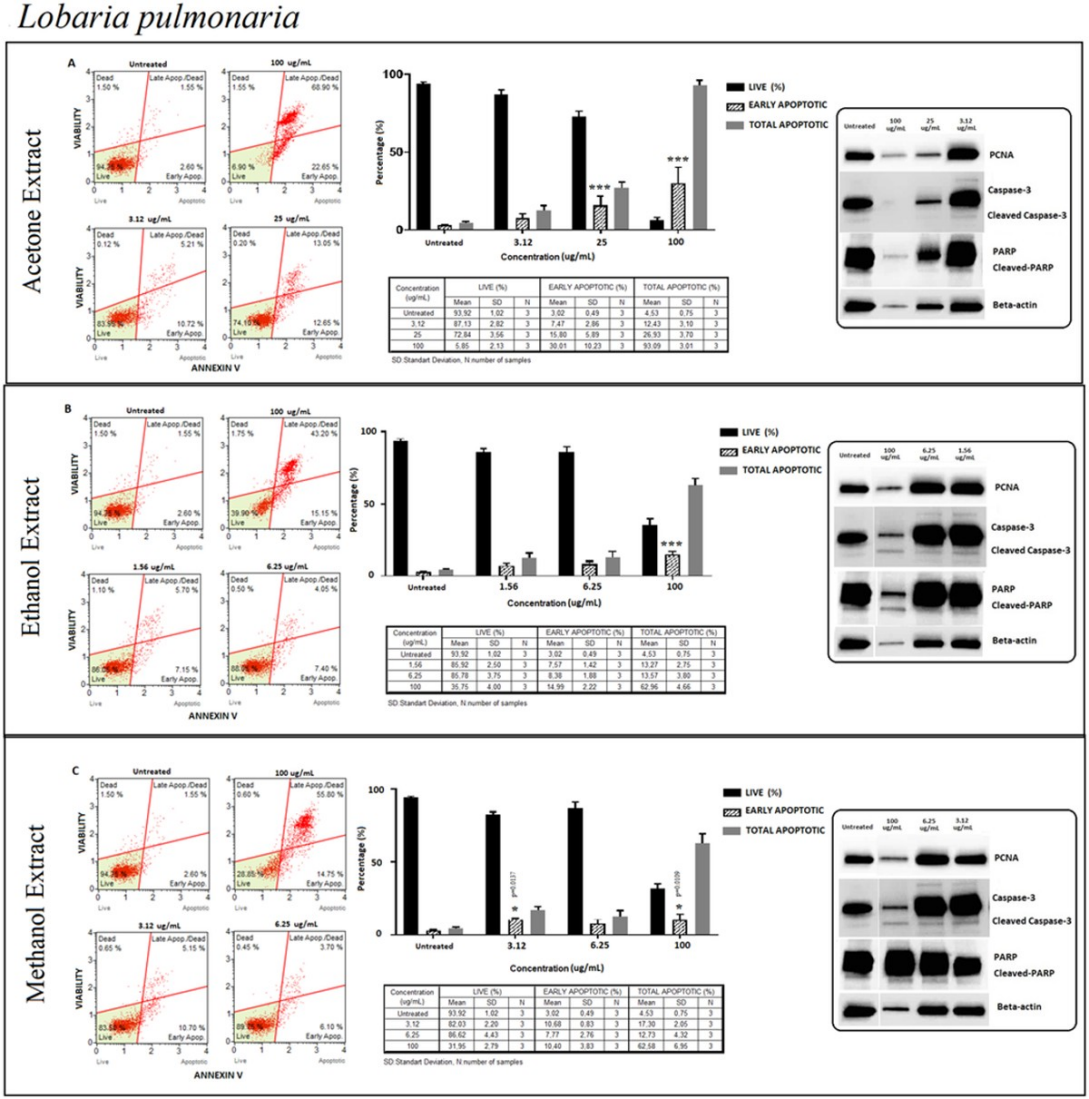
*Lobaria pulmonaria* (LP); Annexin V/7’AAD staining flow cytometry results (scatterplots), statistical analysis (bar graphs), ±SD changes between experiments (tables) and protein levels of PCNA, caspase-3 and PARP by western blot (right panel) on PC-3 cells with three different extraction of acetone (results compiled in A), ethanol (results compiled in B) and methanol (results compiled in C) respectively. Annexin V/7’AAD staining by flow cytometry of LP on PC-3 cells was shown to induce apoptotic cell death by mainly early apoptosis. The gating was adjusted according to the untreated sample and the representative scatterplots were presented as the percentage of cells that were viable (Ann-V^−^ and 7-AAD^−^), early apoptotic (Ann-V^+^ and 7-AAD^−^), late apoptotic (Ann-V^+^ and 7-AAD^−^), and dead (Ann-V^−^ and 7-AAD^+^). Scatterplots are shown for untreated, 100 μg/mL, 25 μg/mL and 3.12 μg/mL after 72 hours treatment with acetone extract (A) and for ethanol extract treatment, groups are untreated, 100 μg/mL, 6.25 μg/mL and 1.56 μg/mL after 72 hours treatment (B) and for methanol extract treatment, groups are untreated, 100 μg/mL, 6.25 μg/mL and 3.12 μg/mL after 72 hours treatment (C) respectively. The 100 μg/mL concentration is determined as toxic dose for all treatment groups. Results were analyzed and presented as the percentage of three independent experiments of live, early apoptotic and total apoptotic after treatment (bar graphs). Flow cytometry was shown to induce apoptotic cell death by mainly early apoptosis. The significance of only early apoptosis for each concentration indicated with an asterisk and the p value was only presented when the significance level of early apoptosis rate between 0.05 and 0.01. ±SD changes between apoptosis assays were indicated as the mean value and SD changes per concentration and per group of live-early apoptotic-total apoptotic separately (table). Western blot results of proliferating cell marker PCNA and apoptosis-related proteins (right panel) were presented. Dose-dependent PCNA, cleavage of caspase-3 and, PARP respectively. Apoptosis rate seems increased significantly by treatment with specified concentrations by flow cytometry however western blot data showed otherwise for cleaved-caspase-3 and PARP respectively when compared with untreated group. In addition, the proliferation (PCNA) status; decreased with higher concentrations of acetone extract, and significantly increased by ethanol extract. Yet methanol extracts remained stable with specified concentration when compared with untreated group. *p < 0.*05*, **p < 0.*001*, ***p < 0.*0001* (all p-values were obtained by two-way ANOVA following Dunnet's multiple comparison test).

Detection of the percentage of apoptotic and live cells was confirmed by activating procaspase 3 and PARP at the same level in both ethanolic and methanolic extraction methods. The percentage of live cells remained high in all concentrations of different extraction methods except 100 μg/ml where live cell percentage decreased sharply specifically in acetone extraction (Fig 9). Apoptosis occurred at a very low level, where proliferation took place dominantly as confirmed by PCNA protein expression in all extraction methods (Fig 9).

#### *Usnea florida* (UF)

**Fig 10:**
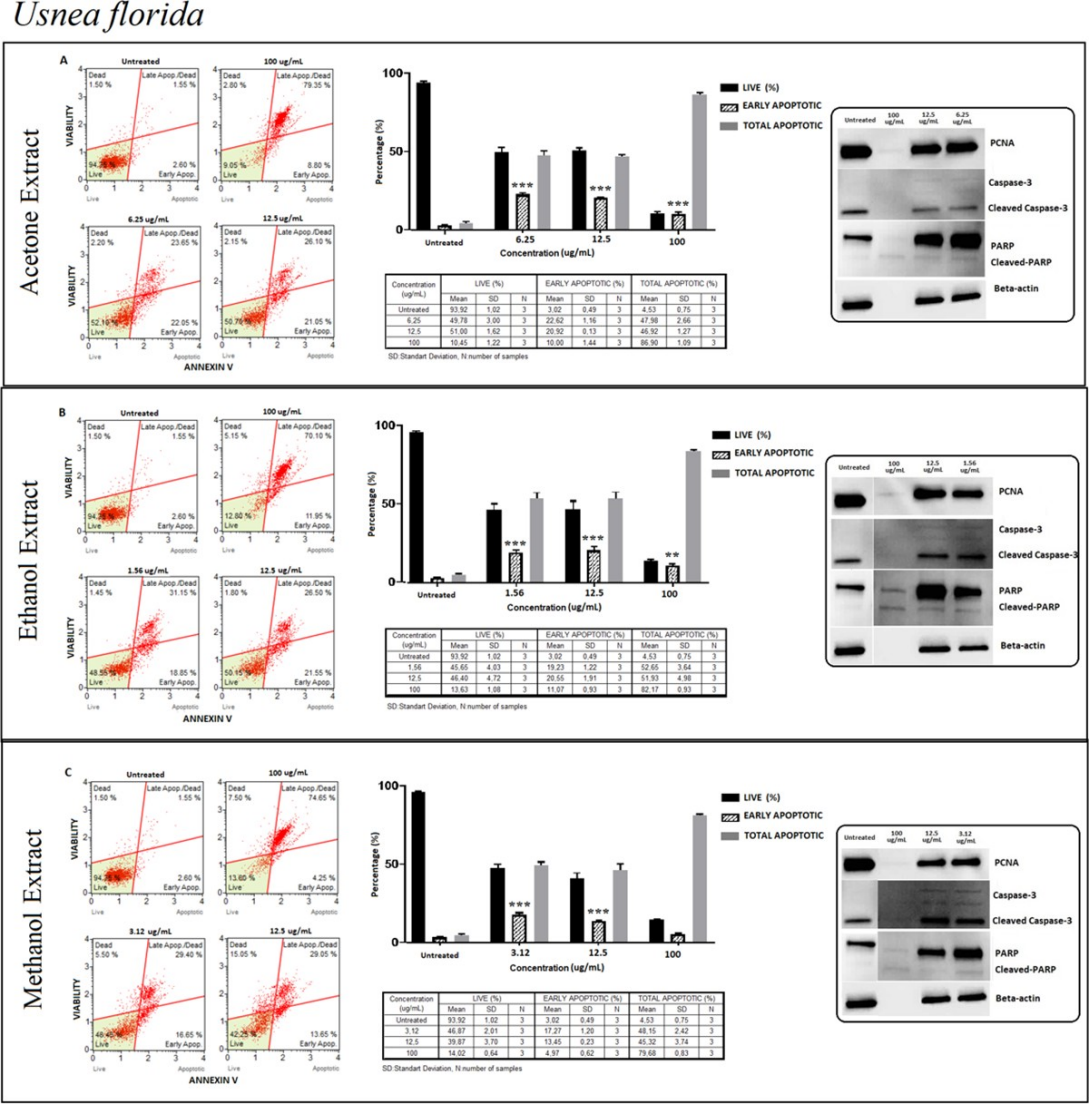
*Usnea florida* (UF); Annexin V/7’AAD staining flow cytometry results (scatterplots), statistical analysis (bar graphs), ±SD changes between experiments (tables) and protein levels of PCNA, caspase-3 and PARP by western blot (right panel) on PC-3 cells with three different extraction of acetone (results compiled in A), ethanol (results compiled in B) and methanol (results compiled in C) respectively. Annexin V/7’AAD staining by flow cytometry of UF on PC-3 cells was shown to induce apoptotic cell death by mainly early apoptosis. The gating was adjusted according to the untreated sample and the representative scatterplots were presented as the percentage of cells that were viable (Ann-V^−^ and 7-AAD^−^), early apoptotic (Ann-V^+^ and 7-AAD^−^), late apoptotic (Ann-V^+^ and 7-AAD^−^), and dead (Ann-V^−^ and 7-AAD^+^). Scatterplots are shown for untreated, 100 μg/mL, 12.5 μg/mL and 6.25 μg/mL after 48 hours treatment with acetone extract (A) and for ethanol extract treatment, groups are untreated, 100 μg/mL, 12.5 μg/mL and 1.56 μg/mL after 48 hours treatment (B) and for methanol extract treatment, groups are untreated, 100 μg/mL, 12.5 μg/mL and 3.12 μg/mL after 48 hours treatment (C) respectively. The 100 μg/mL concentration is determined as toxic dose for all treatment groups. Results were analyzed and presented as the percentage of three independent experiments of live, early apoptotic and total apoptotic after treatment (bar graphs). Flow cytometry was shown to induce apoptotic cell death by mainly early apoptosis. The significance of only early apoptosis for each concentration indicated with an asterisk and the *p* value was only presented when the significance level of early apoptosis rate between 0.05 and 0.01. ±SD changes between apoptosis assays were indicated as the mean value and SD changes per concentration and per group of live-early apoptotic-total apoptotic separately (table). Western blot results of proliferating cell marker PCNA and apoptosis-related proteins (right panel) were presented. Dose-dependent PCNA, cleavage of caspase-3 and, PARP respectively. Apoptosis rate seems increased significantly by treatment with specified concentrations by flow cytometry however western blot data showed otherwise, particularly for cleaved-PARP. In addition, the proliferation (PCNA) status remained stable. *p < 0.*05*, **p < 0.*001*, ***p < 0.*0001* (all p-values were obtained by two-way ANOVA following Dunnet's multiple comparison test).

In all extraction methods that were confirmed by Annexin V assay, the pattern of live and apoptotic cells did not differ in relation to the dose except at 100 μg/ml. The percentage of live cells recorded a 50% decrease and early apoptotic cells remained at 25% in lower concentrations (Fig 10). These results could also be confirmed by PCNA and cleaved caspase 3 expression levels. Early apoptotic cells decreased at 100 μg/ml concentration, while the total apoptotic cells increased sharply indicating a possible role of necrosis in the cell death process (Fig 10).

### Determination of cell proliferation by Western blot analysis

PCNA protein expression levels in each group were determined and changes in the expression profiles of live population were evaluated by Annexin V/7’ADD for each extract as well (Figs 4–6, 8–10).

PCNA density of the three extracts from each BC, CF, LP and, UF methanolic extracts did not change when compared with the untreated group. On the other hand, PCNA density of LP-E was significantly increased (*p = 0*.*0031*) and acted as a proliferating agent but did not have any effects on apoptosis. Besides, LP-A changed PCNA density exhibiting a dose-dependent manner, however, increasing the dose did not correlate with the apoptotic behavior in Annexin V assay results. Neither 25 μg/mL nor 3.12 μg/mL of extract dose enhanced apoptosis as seen in cleaved caspase-3 and cleaved-PARP (Figs 4–6, 8–10). Therefore, the outcome positively correlated with Annexin V results.

## Discussion

Lichens are one of the most valuable plant resources that are utilized in different areas including medicine [27]. Whole extracts or secondary metabolites of lichens have anticancer properties [28]. In this study, anti-proliferative and apoptotic effects of ethanol, methanol, and acetone extracts of *Bryoria capillaris*, *Cladonia fimbriata*, *Evernia divaricata*, *Hypogymnia tubulosa*, *Lobaria pulmonaria*, *Usnea florida* were investigated in PC-3 human prostate cancer cell line. Although limited studies about lichens demonstrated variable effects on prostate cancer cells *in vitro*, only in one study, anti-proliferative effect of lichen derived lipoxygenase inhibitors on twelve human cancer cell lines including PC-3 cells was reported [15, 29–31]. The lack of studies about apoptosis, proliferation, and cytotoxicity activities on these lichen species in cancer cells renders it difficult to compare our results. In this context, this study is the first report of most of the lichen species having cytotoxic and apoptotic effects on PC-3 cells.

Lichens reportedly exhibit cytotoxic activity in different cancer cell lines [28, 32] and we have discovered that lichen extracts exhibit different levels of cytotoxic ability depending on the treatment dose and time. Accordingly, anti-proliferative and apoptosis-inducing effects of BC were investigated eminently revealing that early necrosis might have been observed at 100 μg/ml. and slight apoptotic effects could only be observed at lower doses. As far as we are concerned, our study is the first to report on the apoptotic effects of *Bryoria capillaris*, therefore a comparison of results could not be performed for this lichen species. However, *Bryoria capillaris* extracts have been shown in various studies to have anti-proliferative effects on breast and lung cancer cells in a dose-dependent manner [33], which is also supported by our study.

Various studies on Cladonia species have been shown to have cytotoxic, anti-proliferative, and apoptotic effects in certain cancer cell lines [30, 34–36]. Mitrović *et al*. reported anti-proliferative activities of the five lichens species *Parmelia sulcata*, *Flavoparmelia caperata*, *Evernia prunastri*, *Hypogymnia physodes*, and *Cladonia foliacea* [37]. Another study indicated the methanolic extraction of two Cladonia species have cytotoxic and strong anti-proliferative effects on MCF-7 cells [34]. The present study shows only up to 70% cytotoxic activity in all three extracts evaluated by MTT. It was observed that PCNA protein expression decreased insignificantly in three extracts of *Cladonia fimbriata*, which conflicts with other studies. Environmental factors including temperature, seasonality, UV-exposure, elevation as well as age of lichens influence secondary metabolites [38]. Therefore, it is safe to assume that the contradicting results could be caused by various other factors such as the type of cancer cell line.

As far as the literature concerned, our study on *Evernia divaricata* induced anti-proliferative and apoptotic effects in PC-3 cells is the first report presented at the time of this study. However, anti-proliferative and cytotoxic effects of *E*. *prunastri* which is a different species of Evernia has been demonstrated in several studies [39, 40] and is described in the literature for their usnic acid content [39]. We investigated increased cytotoxicity levels especially in acetonic extracts and usnic acid is likely to be involved in the cytotoxic activities observed. A study done by Yumrutaş *et al*. that investigated the antioxidant activity of methanol extract of five lichens in Turkey reported that the extracts of *Evernia divaricata* did not exert any activity [40]. Cell viability reached peak levels at the lowest concentrations in our study, which is a widely observed antioxidant effect pattern. Consequently, acetone extraction of *Evernia divaricata* might specifically reveal antioxidant effects. Potential antioxidant activities of phenolic components have been shown previously [41] and it is possible by acetone extraction, these phenolic contents might have emerged. Our data indicate that *Evernia divaricata* induced apoptotic cell death via both extrinsic and intrinsic pathways. By caspase 9 activation cells, undergo apoptosis or it was also shown that this protein has a role in autophagy [42]. This finding by Jeong HY *et al*. is consistent with our findings where ethanol extraction of *Evernia divaricata* induced the same percentages of apoptotic cells as live cells decreased sharply.

Further, this study is the first attempt to demonstrate the apoptotic effects and anti-proliferative activity of *Hypogymnia tubulosa* extracts on prostate cancer cells in which we established both the percentage of the apoptotic cells and apoptotic protein expressions are increased at lower treatment concentrations. At the highest treatment dose, only very low level of apoptotic cells and apoptotic protein markers but significantly increased rate of necrotic cells were reported.

However, secondary metabolites derived from different species of Hypogymnia exhibit these effects [12, 37, 43, 44] which is consistent with our study. Extracts of *Hypogymnia tubulosa* exhibited cell viability reduction in a dose-dependent manner. Hence, for a longer time of treatment exposure, a higher cell sensitivity is monitored, excluding the lower concentrations of the extract. The comparison of the cytotoxicity at three-time intervals revealed a time-dependent increase of cytotoxicity for higher concentrations. Our results indicated that the higher the treatment dose of the extract applied, the higher the cell sensitivity observed. Also, the longer the time of the treatment, the higher the cell sensitivity recorded.

Our results on *Lobaria pulmonaria* extracts are correlated with various studies. Shresta *et al*. treated Raji cells with LP acetone extracts from 17 different areas of North America and evaluated IC50 values [45]. In addition, *Lobaria pulmonaria* methanolic extract was reported to have a higher phenolic content, antioxidant activity, and reducing powers than *U*. *longissima*, *U*. *florida* [46]. Another study demonstrated that *Lobaria pulmonaria* has anti-proliferative effect on breast and lung cancer cells in a dose-dependent manner [33]. In our findings, prostate cancer cells exhibited significant cytotoxic effects in a dose and time-dependent manner in all three extraction methods. However, based on the apoptotic cell rates and apoptotic protein markers, we could speculate that cell death occurs through necrosis.

Various studies reported different effects and outcomes for Usnea species including anti-proliferative and antioxidant properties [31, 45, 46]. Galanty *et al*. reported that usnic acid, one of the secondary metabolites of Usnea inhibits proliferation and induces apoptosis in PC-3 cells while displaying relatively neutral to normal cells. Our results are consistent with this study which we have demonstrated that apoptosis induction occurs at lower concentration of extract treatments. At the highest concentration of extracts, apoptotic cell rates as well as apoptotic protein expression levels reduced. As shown in the flow cytometry results, late apoptotic or secondary necrotic cell percentages elevated, which is confirmed by the increased cytotoxicity percentages at the highest extract concentration.

In our study, the best solvent to obtain anti-proliferative effect of lichen extracts was determined. Acetone, ethanol, and methanol extraction methods of *Evernia divaricata*, *Hypogymnia tubulosa* showed significant apoptotic activity on prostate cancer cells even at low concentrations. Especially, *Evernia divaricata* -A extract had the greatest apoptotic behavior on prostate cancer cells when compared to the other five lichen species with three extraction methods. Annexin V/7’AAD staining was also correlated with an apoptotic outcome. Prolonged activation of the apoptotic cascade leads to the enhancement of a beneficial response on prostate cancer cells. Specifically, acetone extraction of *Evernia divaricata* without depending on the dose and acetone, ethanol, methanol extracts of HT with a low to a higher impact when considered only as a cleavage product of PARP was determined. We evaluated cleaved caspase-8 and cleaved caspase-9 only in *Evernia divaricata*, *Hypogymnia tubulosa* extracts where the activation level of cleaved-PARP was more significant. Even though both natural extracts converged towards mitochondrial collapse, the induction of apoptosis was possibly extract specific. The results we obtained will need further analysis to determine secondary metabolites in effective lichen extracts.

Our study had several limitations including the comparison of different extraction types of same lichen species due to the variations in the concentration of doses and incubation times. Another limitation was regarding the dynamic structures of cell lines that differ in genotypes and phenotypes where only PC3 androgen-independent cancer cell lines were used. We believe further studies are needed on different prostate cancer cells, androgen-dependent prostate cancer cells, and non-malignant cells to reveal relatively more definitive outcomes.

Based on our findings, we conclude that increased efforts in screening natural products as potential sources for future anticancer treatments should be a priority. We tested 18 extracts of six lichen species during our study. Of these, *Evernia divaricata* -A and *Hypogymnia tubulosa* demonstrated significant apoptotic activity on prostate cancer cells including at low concentrations, which implies that it is worth pursuing the biologically active lead compounds of these extracts on prostate cancer *in vitro*. Further corroboratory studies are needed to validate the relative potential of these extracts as anti-metastatic and anti-tumorigenic agents.

## Supporting information

S1 File Raw images of Western blots. (PDF)

S2 File Raw flow cytometry results of each lichen species. (PDF)

S3 File Apoptosis analysis file of each lichen species. (PZF)

## Abbreviations

7-AAD: 7-amino actinomycin
BC: *Bryoria capillaris*
CF: *Cladonia fimbriata*
DMSO: Dimethyl sulphoxide
ECL: Enhanced chemiluminescence
ED: *Evernia divaricata*
FBS: Fetal bovine serum
HT: *Hypogymnia tubulosa*
LDH: Lactate dehydrogenase
LP: *Lobaria pulmonaria*
MTT: 4,5-dimethylthiazol-2-yl)-2,5 diphenyltetrazolium bromide
NAD+: Nicotinamide adenine dinucleotide
PARP: Poly (ADP-ribose) polymerase
PC-3: Human androgen-independent prostate cancer cells
PCa: Prostate Cancer
PCNA: Proliferating Cell Nuclear Antigen
PCNA: Proliferating cell nuclear antigen
PVDF: polyvinylidene difluoride
RIPA lysis buffer: Radioimmunoprecipitation assay lysis buffer
RPMI 1640: Roswell Park Memorial Institute 1640 medium
TAGEM: Republic of Turkey Ministery Agriculture and Forestry
TBST: Tris Buffered Saline with 0.02% Tween-20
UF: *Usnea florida*
