# Correction: Differential anti-proliferative and apoptotic effects of lichen species on human prostate carcinoma cells

**DOI:** 10.1371/journal.pone.0244831

**Published:** 2020-12-28

**Authors:** Beyza Goncu, Ece Sevgi, Cagla Kizilarslan Hancer, Guzin Gokay, Nur Ozten

The ORCID iD is missing for the second author. Please see the author’s ORCID iD here:

- Author Ece Sevgi’s ORCID iD is: 0000-0002-8247-5178 (https://orcid.org/0000-0002-8247-5178).
