# Supplementary material for: Differential anti-proliferative and apoptotic effects of lichen species on human prostate carcinoma cells
Source: PLoS One. 2020 Sep 30;15(9):e0238303. doi: 10.1371/journal.pone.0238303 (PMC7527208; doi:10.1371/journal.pone.0238303)
Supplement: S1 File — (PDF) [file pone.0238303.s001.pdf]

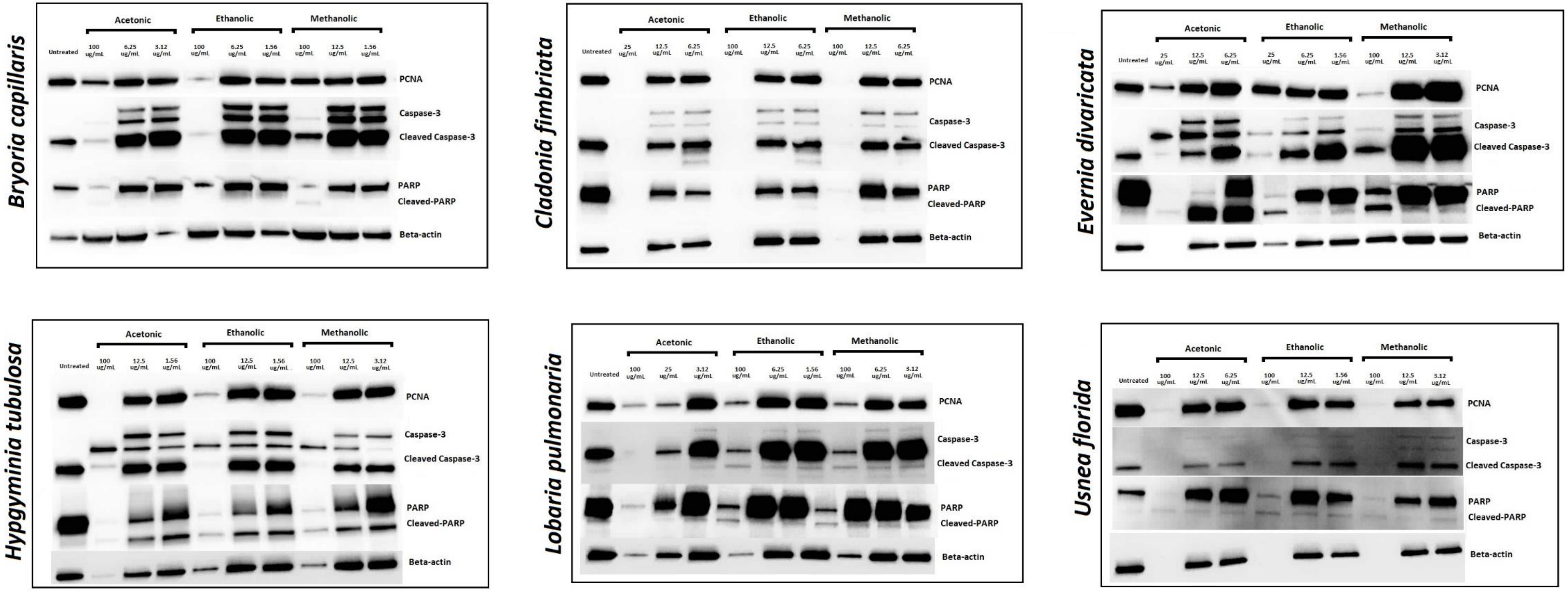
Bryoria capillaris
Acetonic
Ethanolic
Methanolic
Untreated
100 ug/mL
6.25 ug/mL
3.12 ug/mL
100 ug/mL
6.25 ug/mL
1.56 ug/mL
100 ug/mL
12.5 ug/mL
1.56 ug/mL
PCNA
Caspase-3
Cleaved Caspase-3
PARP
Cleaved-PARP
Beta-actin
Cladonia fimbriata
Acetonic
Ethanolic
Methanolic
Untreated
25 ug/mL
12.5 ug/mL
6.25 ug/mL
100 ug/mL
12.5 ug/mL
6.25 ug/mL
100 ug/mL
12.5 ug/mL
6.25 ug/mL
PCNA
Caspase-3
Cleaved Caspase-3
PARP
Cleaved-PARP
Beta-actin
Evernia divaricata
Acetonic
Ethanolic
Methanolic
Untreated
25 ug/mL
12.5 ug/mL
6.25 ug/mL
25 ug/mL
6.25 ug/mL
1.56 ug/mL
100 ug/mL
12.5 ug/mL
3.12 ug/mL
PCNA
Caspase-3
Cleaved Caspase-3
PARP
Cleaved-PARP
Beta-actin
Hypgyminia tubulosa
Acetonic
Ethanolic
Methanolic
Untreated
100 ug/mL
12.5 ug/mL
1.56 ug/mL
100 ug/mL
12.5 ug/mL
1.56 ug/mL
100 ug/mL
12.5 ug/mL
3.12 ug/mL
PCNA
Caspase-3
Cleaved Caspase-3
PARP
Cleaved-PARP
Beta-actin
Lobaria pulmonaria
Acetonic
Ethanolic
Methanolic
Untreated
100 ug/mL
25 ug/mL
3.12 ug/mL
100 ug/mL
6.25 ug/mL
1.56 ug/mL
100 ug/mL
6.25 ug/mL
3.12 ug/mL
PCNA
Caspase-3
Cleaved Caspase-3
PARP
Cleaved-PARP
Beta-actin
Usnea florida
Acetonic
Ethanolic
Methanolic
Untreated
100 ug/mL
12.5 ug/mL
6.25 ug/mL
100 ug/mL
12.5 ug/mL
1.56 ug/mL
100 ug/mL
12.5 ug/mL
3.12 ug/mL
PCNA
Caspase-3
Cleaved Caspase-3
PARP
Cleaved-PARP
Beta-actin

BC- extraction

Well Order	Ladder-Control-A100-A6.25-A1.56-E100-E6.25-E1.56-M100-M12.5-M1.56<br>(A indicates Acetonic. E indicates Ethanolic. M indicates Methanolic)
Beta-aktin (retrieved from 1sec exposure)	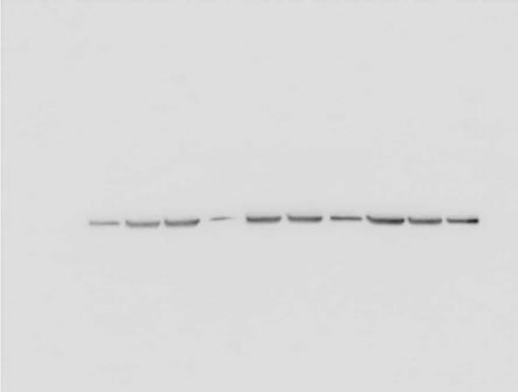 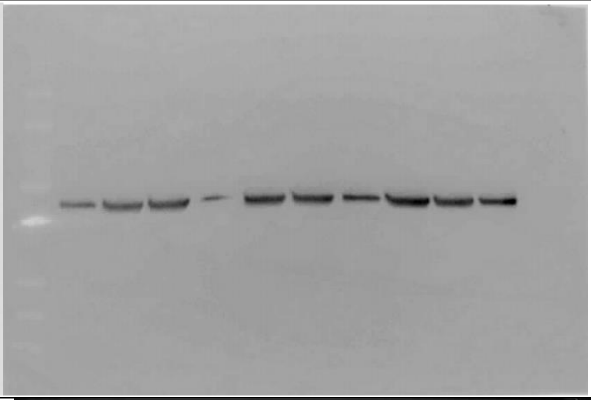
Beta-aktin (retrieved from 10sec exposure)	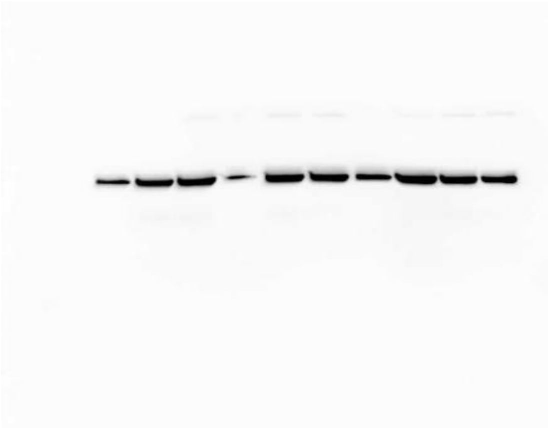 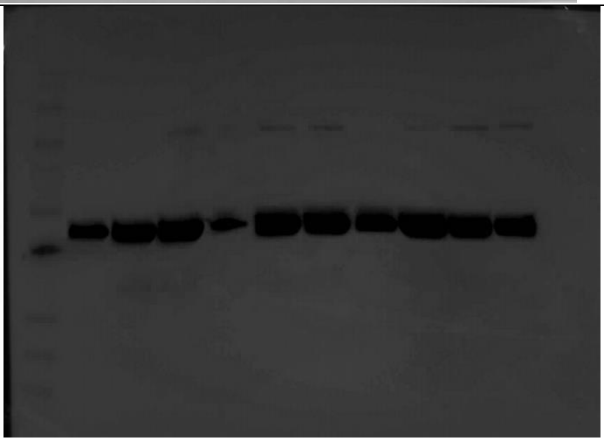
Beta-aktin (retrieved from 3min exposure)	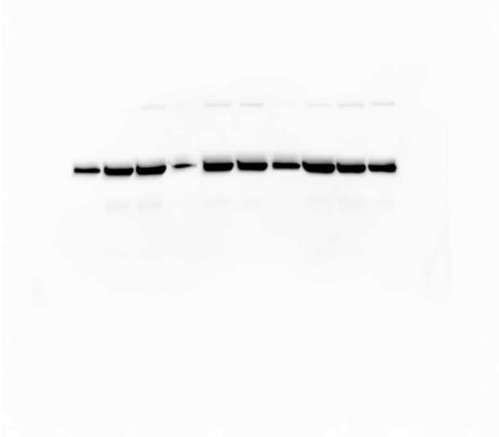 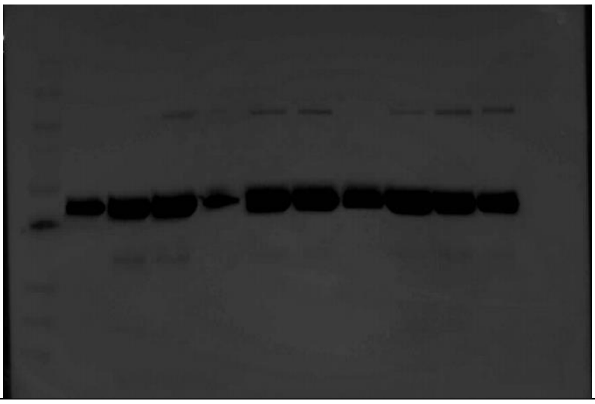

PCNA (retrieved from 1sec exposure)	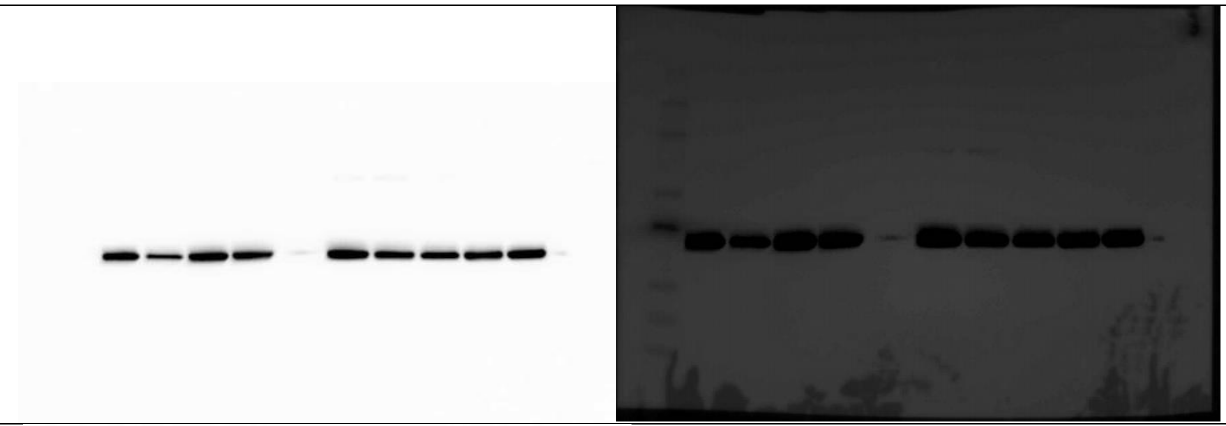
Caspase-3 (retrieved from 3min exposure)	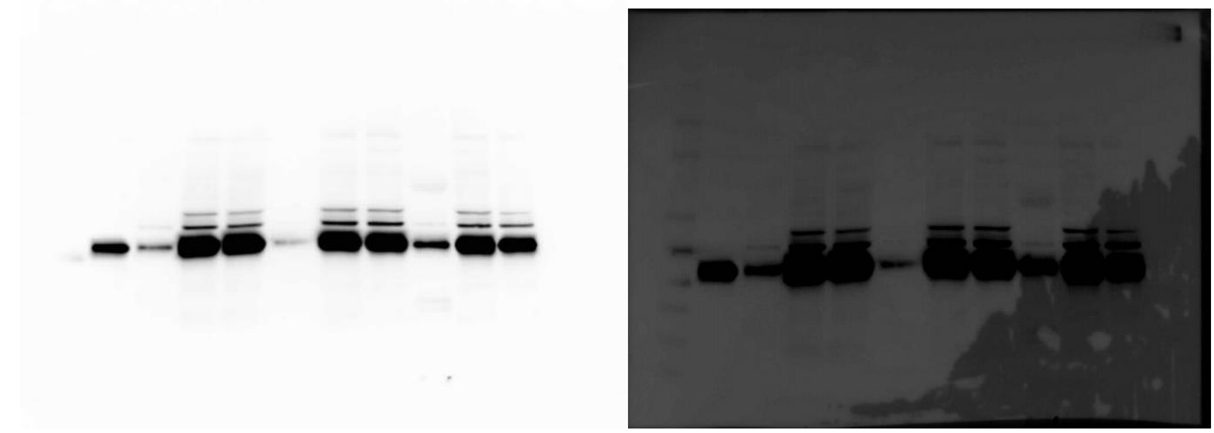
PARP (retrieved from 10sec exposure)	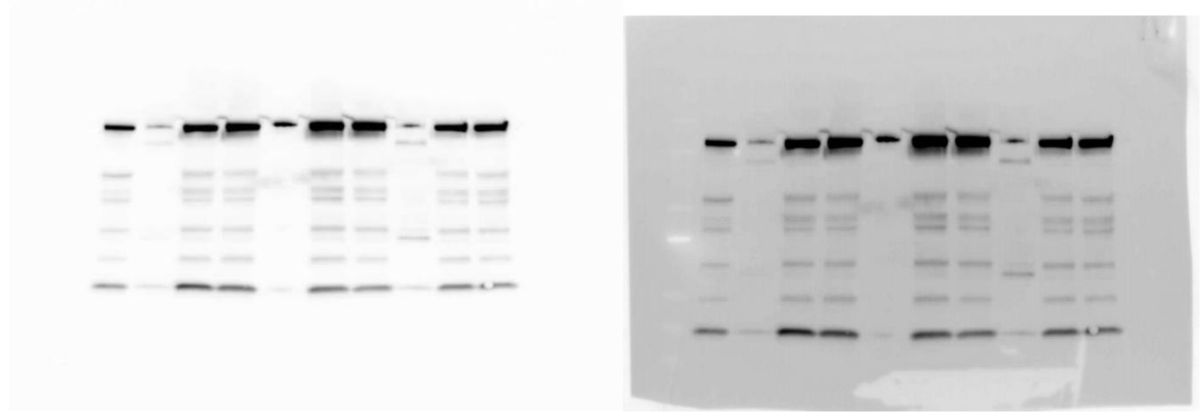

CF-extraction

Well Order	Ladder-Control-A100-A12.5-A6.25-E100-E-12.5-E6.25-M100-M12.5-M6.25<br>(A indicates Acetonic. E indicates Ethanolic. M indicates Methanolic)
Beta-aktin (retrieved from 3sec exposure)	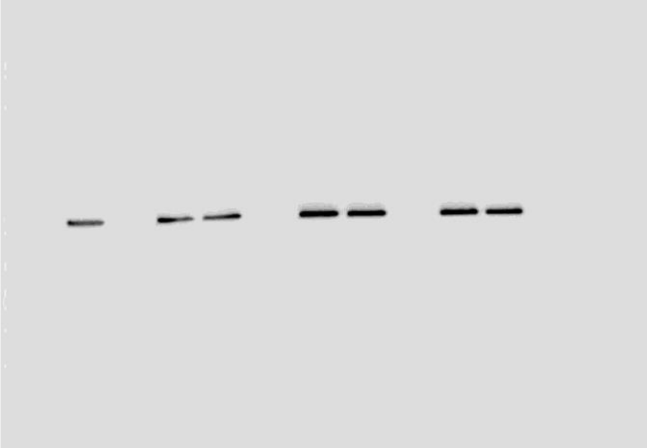 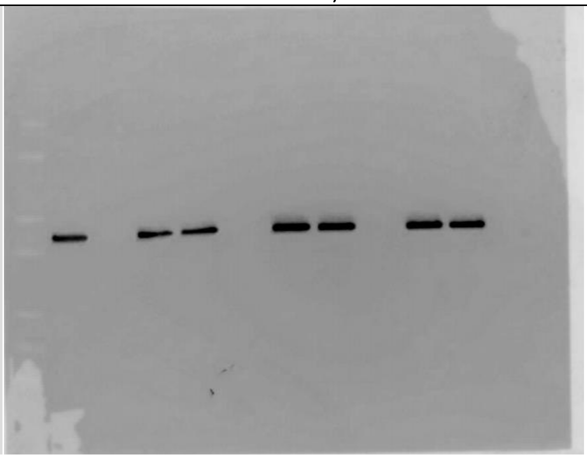
Beta-aktin (retrieved from 10sec exposure)	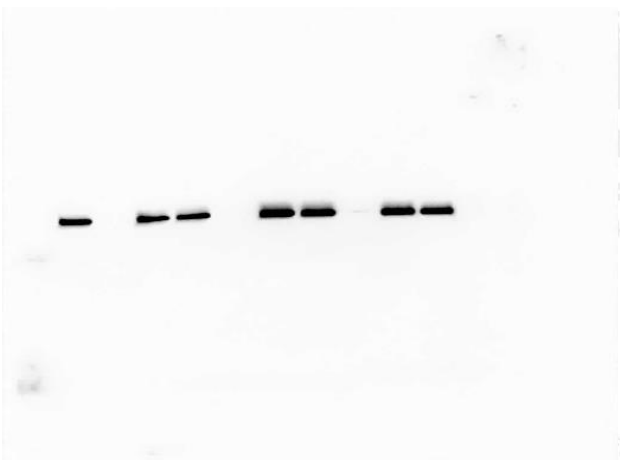 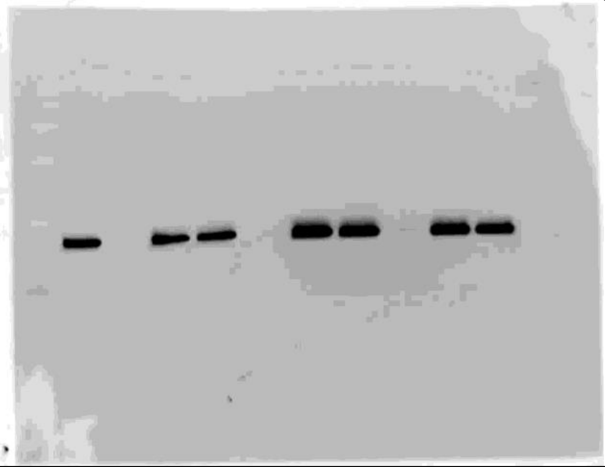
Beta-aktin (retrieved from 45sec exposure)	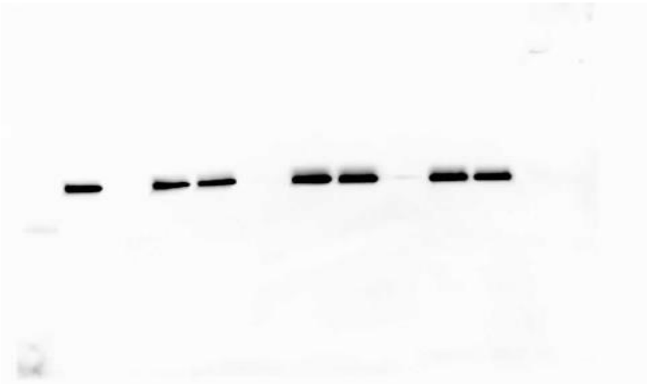 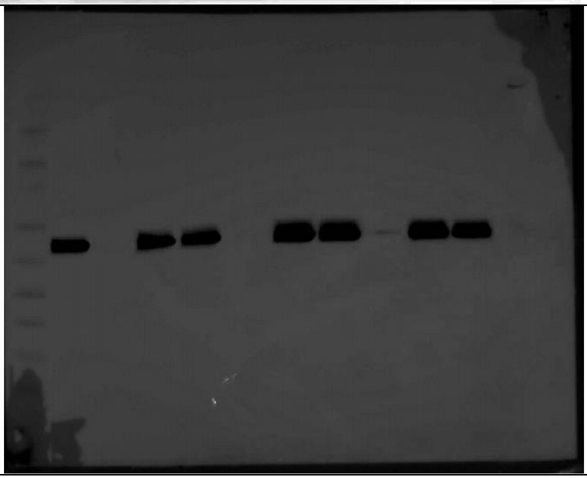

PCNA (retrieved from 3sec exposure)	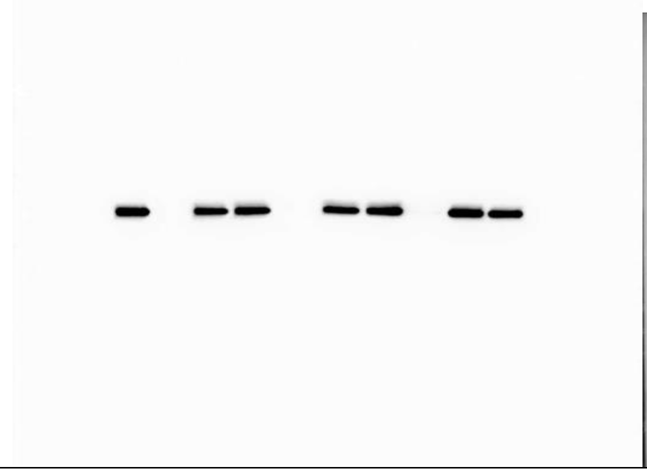 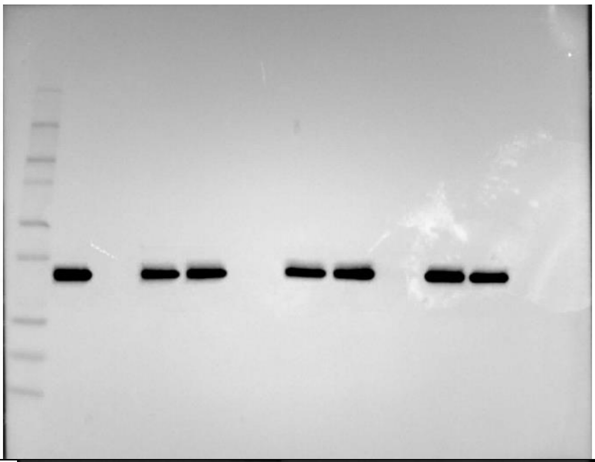
Caspase-3 (retrieved from 45sec exposure)	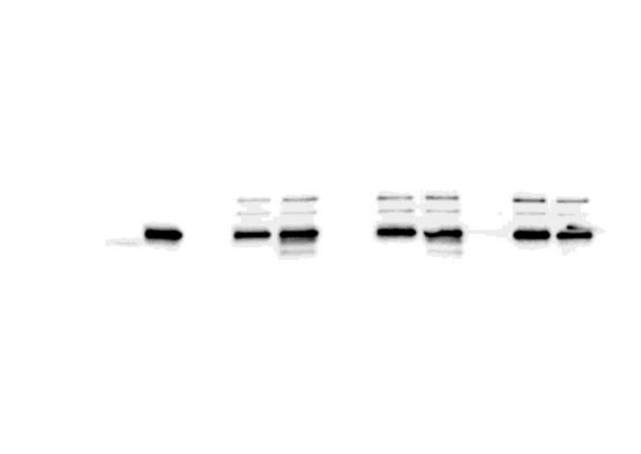 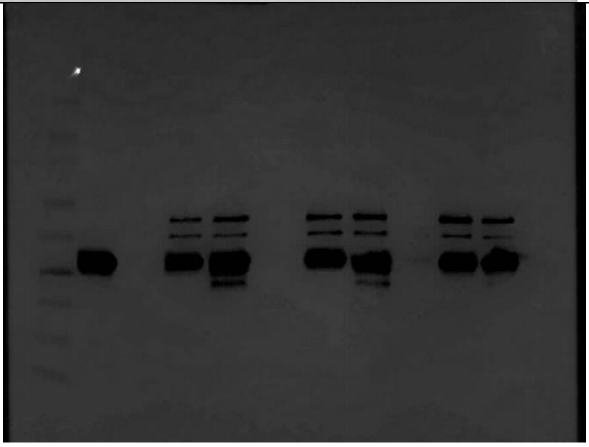
PARP (retrieved from 10sec exposure)	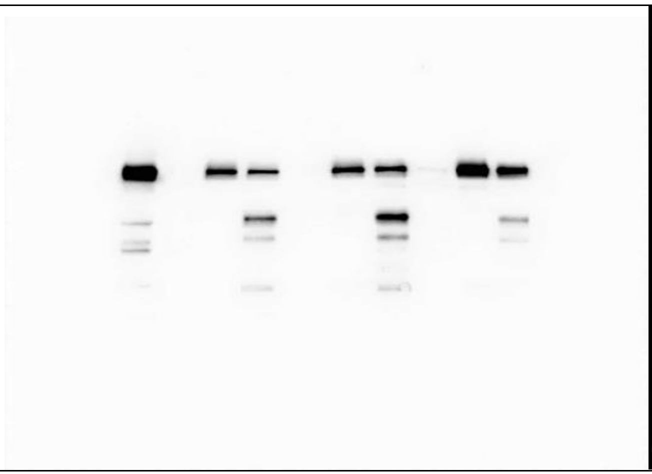 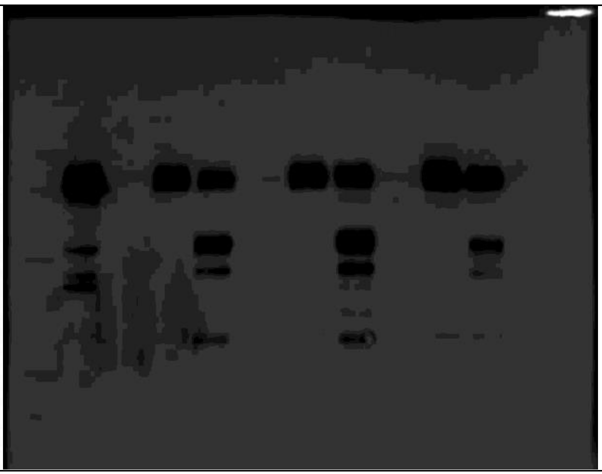

ED-A extraction

Well Order	A25-A12.5-A6.25-Ladder<br>(A indicates Acetonic)
Beta-aktin (retrieved from 8sec exposure)	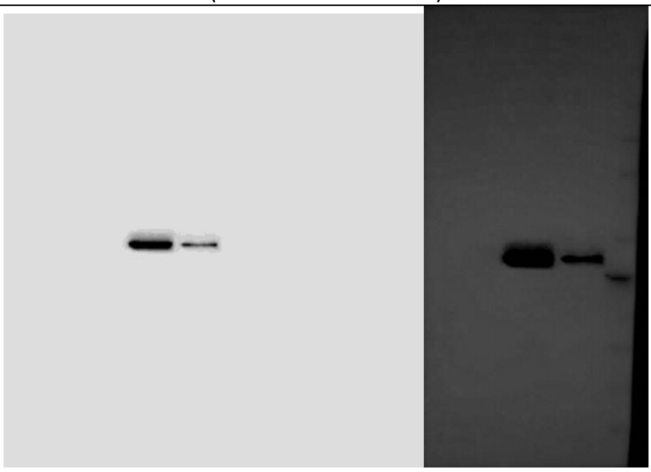
Beta-aktin (retrieved from 3min exposure)	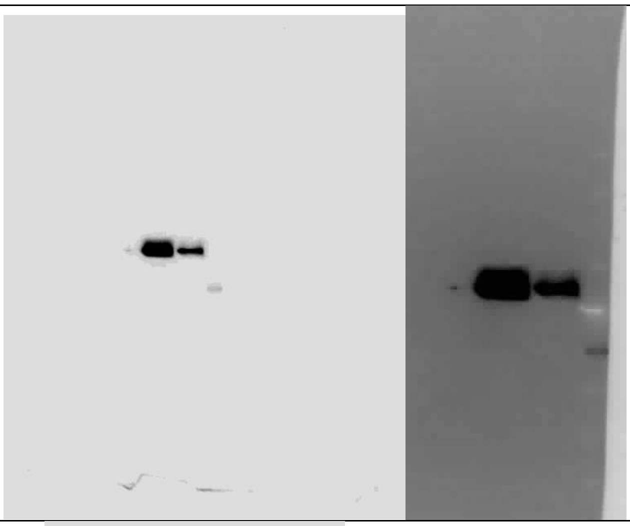
PCNA (retrieved from 5sec exposure)	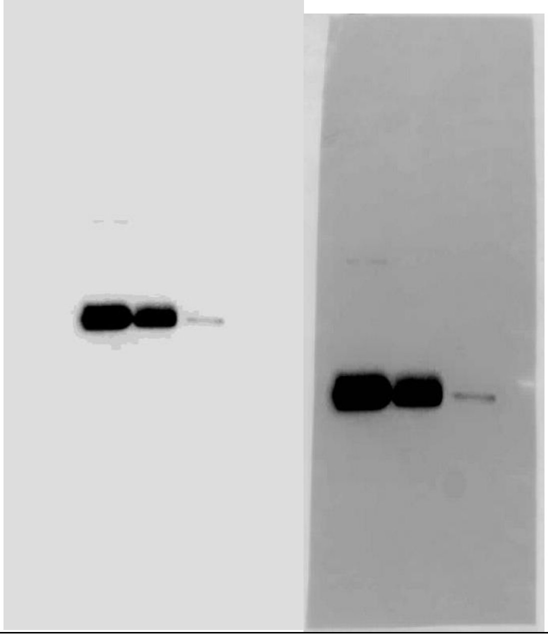

Well order	Ladder-Control-A25-A12.5-A6.25<br>(E indicates Ethanolic. M indicates Methanolic)
Caspase-3 (retrieved from 3min exposure)	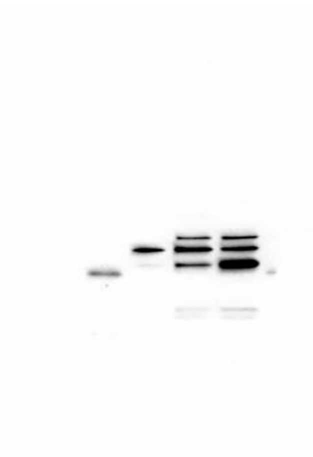 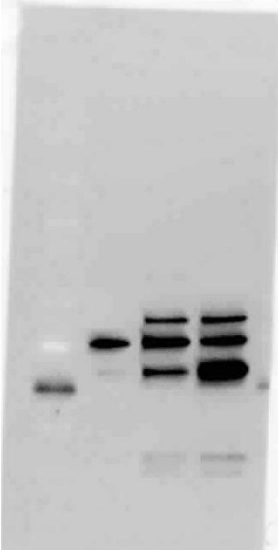
PARP (retrieved from 8sec exposure)	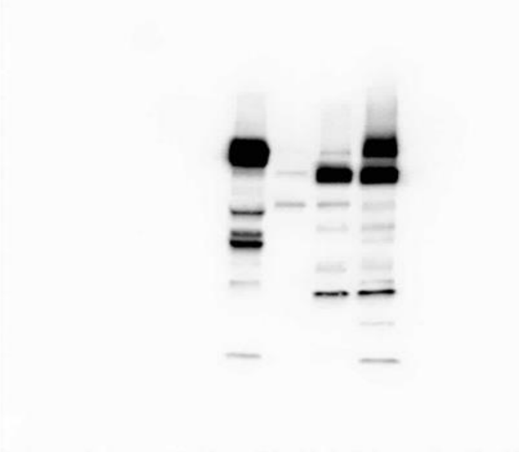 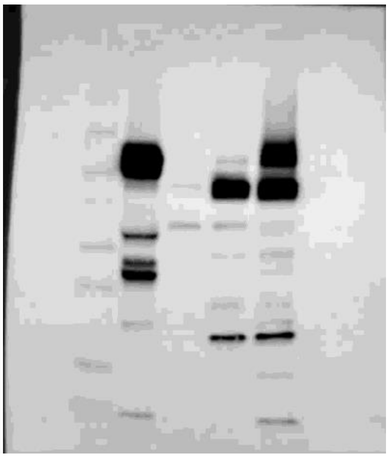

ED-E and ED-M extraction

Well Order	Ladder-Control-E25-E6.25-E1.56-M100-M12.5-M3.12<br>(A indicates Acetonic. E indicates Ethanolic. M indicates Methanolic)
Beta-aktin (retrieved from 1sec exposure)	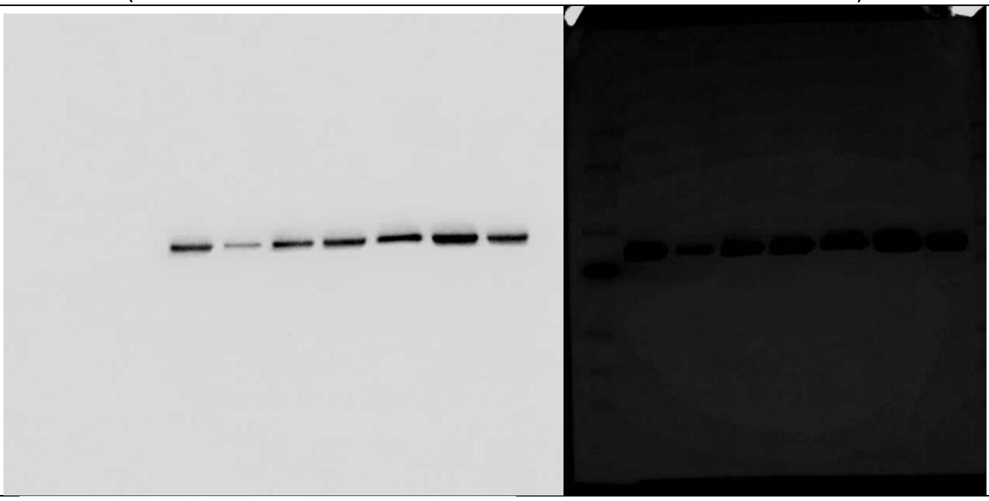
Beta-aktin (retrieved from 10sec exposure)	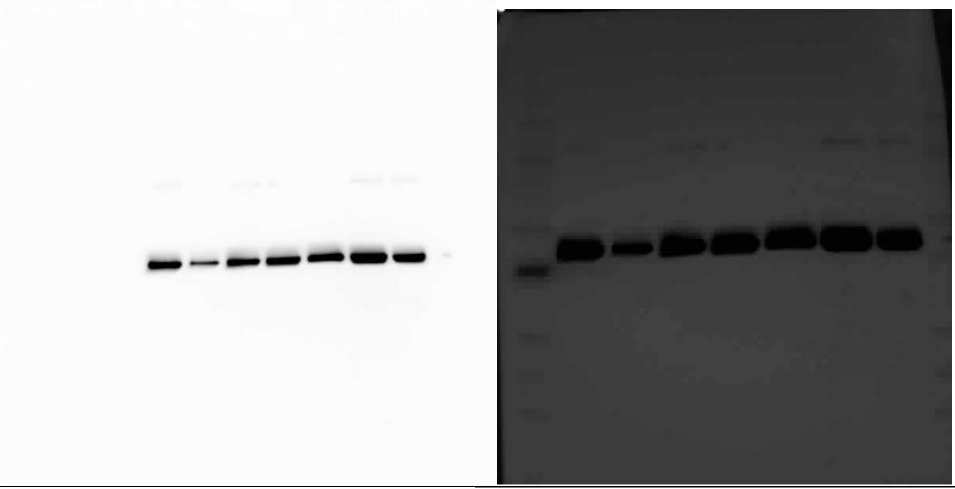
PCNA (retrieved from 1sec exposure)	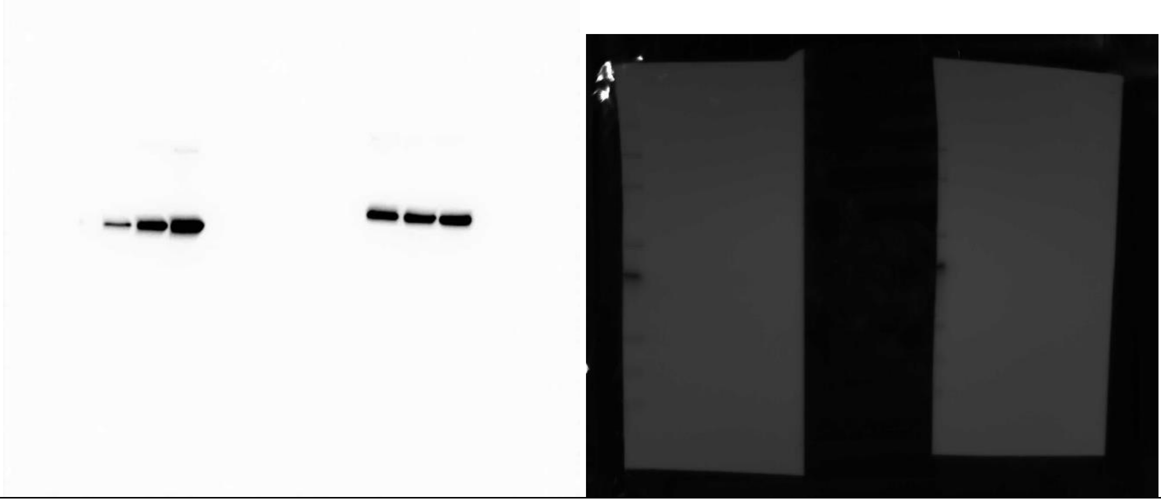

Caspase-3 (retrieved from 10sec exposure)	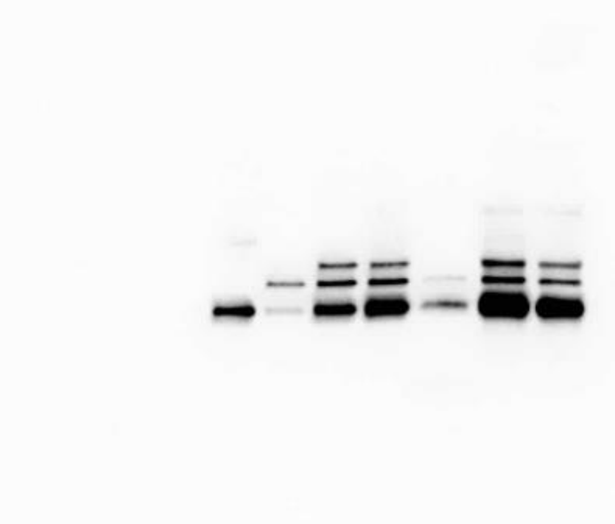 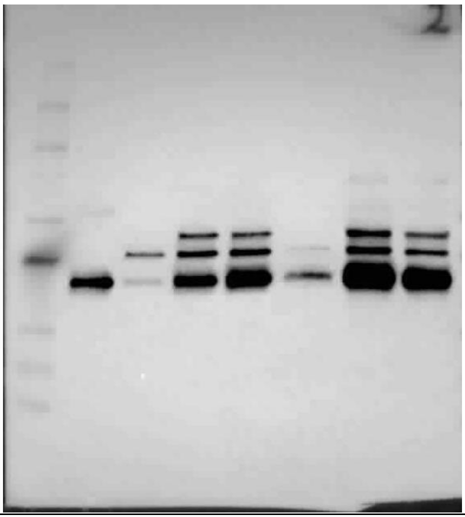
PARP (retrieved from 10sec exposure)	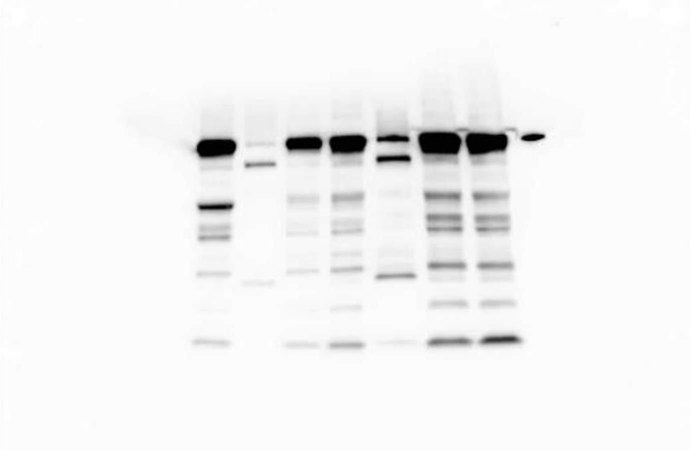 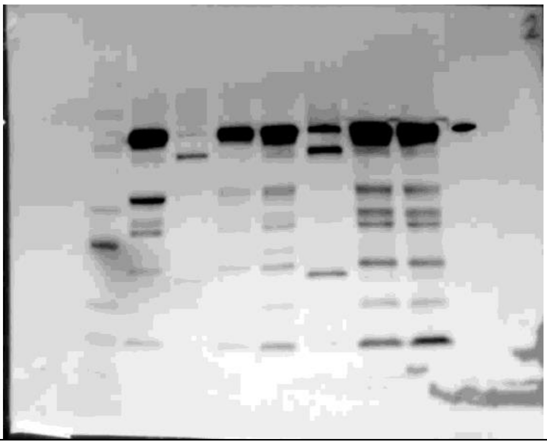

HT-extraction

Well Order	Ladder-Control-A100-A12.5-1.56-E100-E12.5-E1.56-M100-M12.5-M3.12<br>(A indicates Acetonic. E indicates Ethanolic. M indicates Methanolic)
Beta-aktin (retrieved from 5sec exposure)	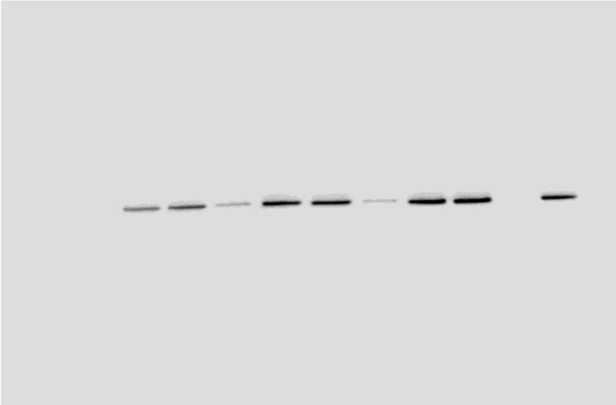 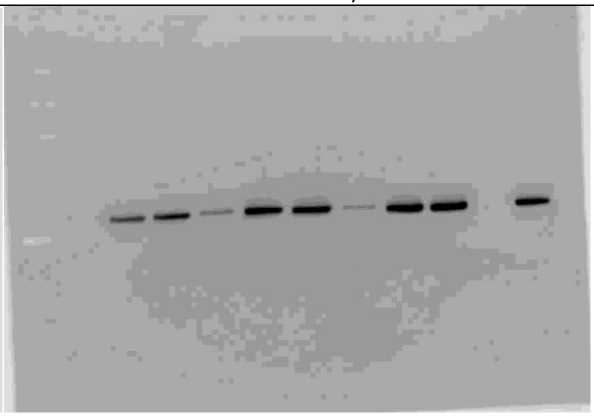
Beta-aktin (retrieved from 10sec exposure)	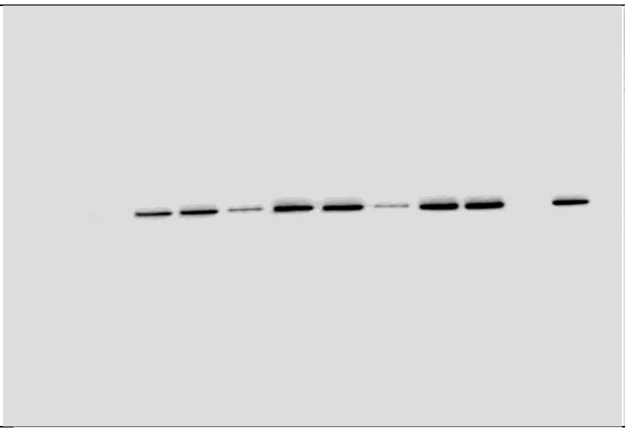 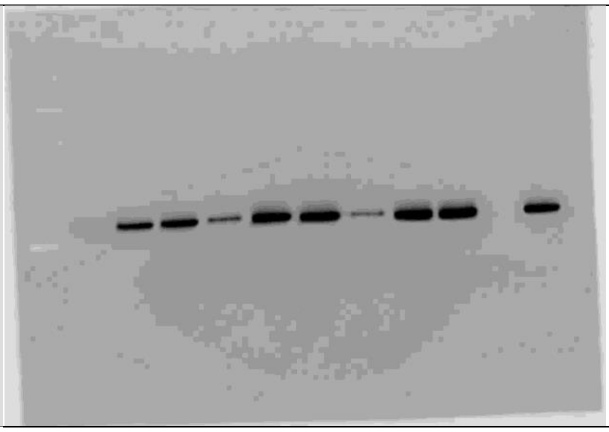
Beta-aktin (retrieved from 3min exposure)	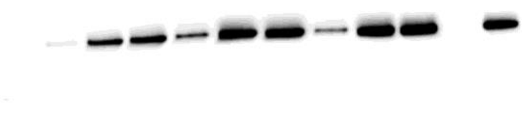 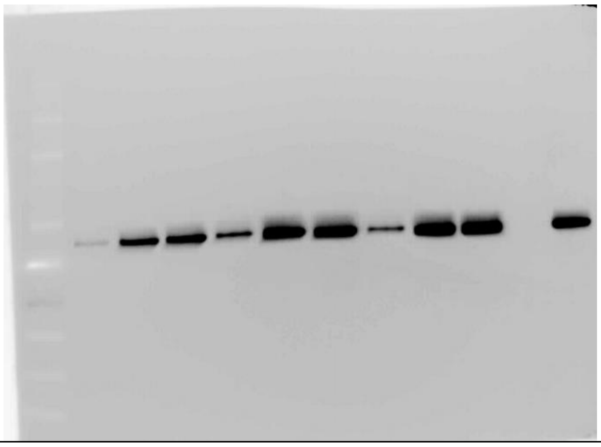

PCNA (retrieved from 2sec exposure)	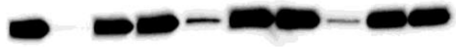 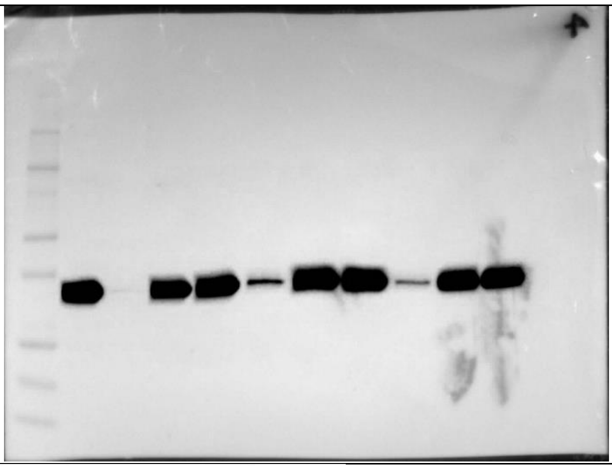
Caspase-3 (retrieved from 3min exposure)	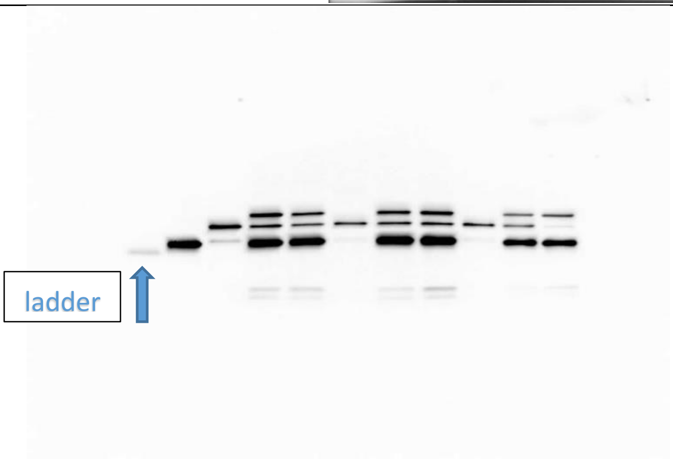
PARP (retrieved from 10sec exposure)	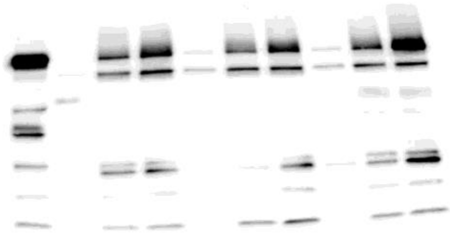 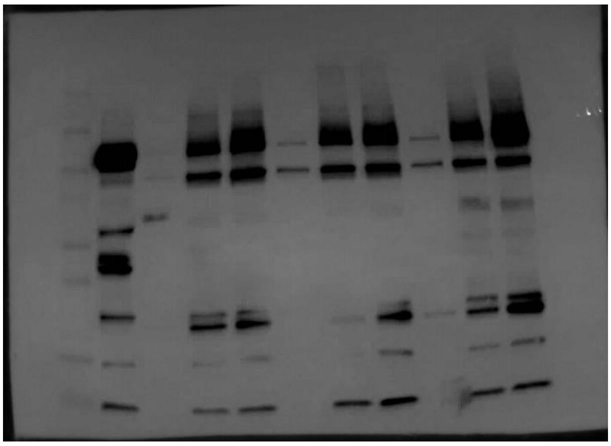

LP-extraction

Well Order	Ladder-Control-A100-A25-A3.12-E100-E6.25-E1.56-M100-M6.25-M1.56<br>(A indicates Acetonic. E indicates Ethanolic. M indicates Methanolic)
Beta-aktin (retrieved from 1sec exposure)	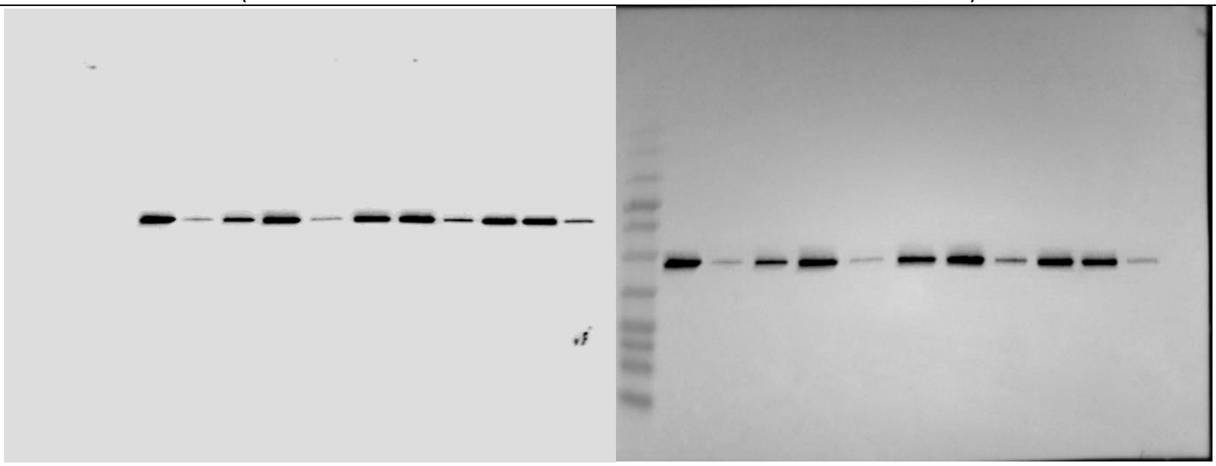
Beta-aktin (retrieved from 3sec exposure)	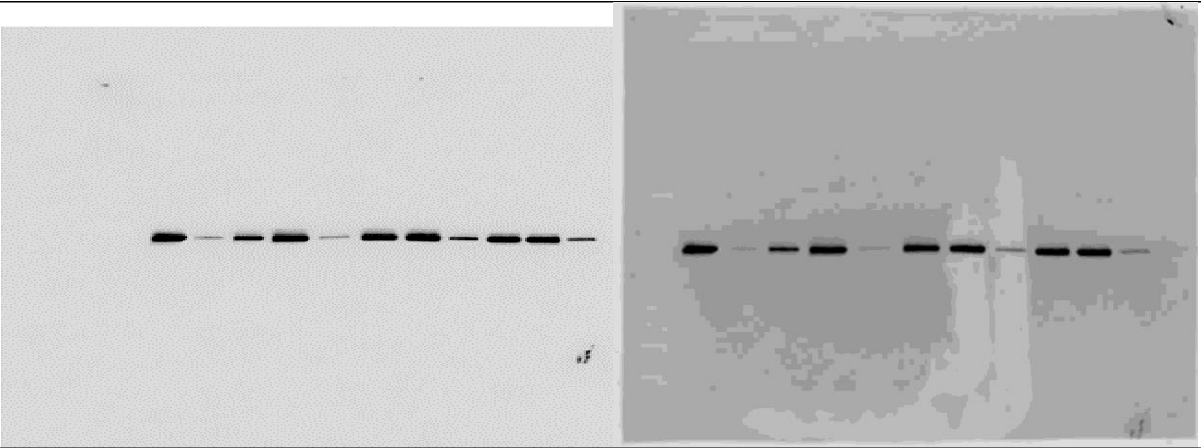
Beta-aktin (retrieved from 10sec exposure)	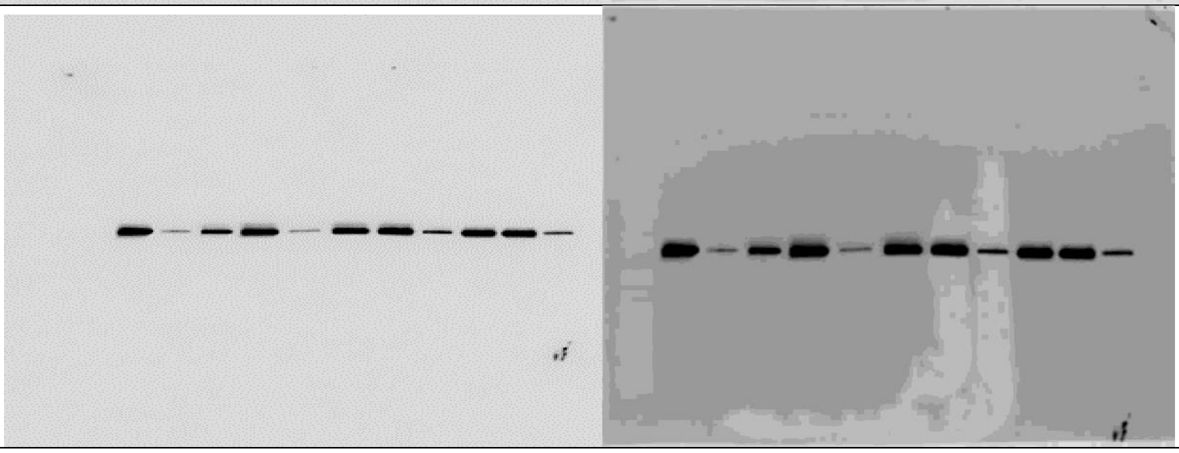

PCNA (retrieved from 1sec exposure)	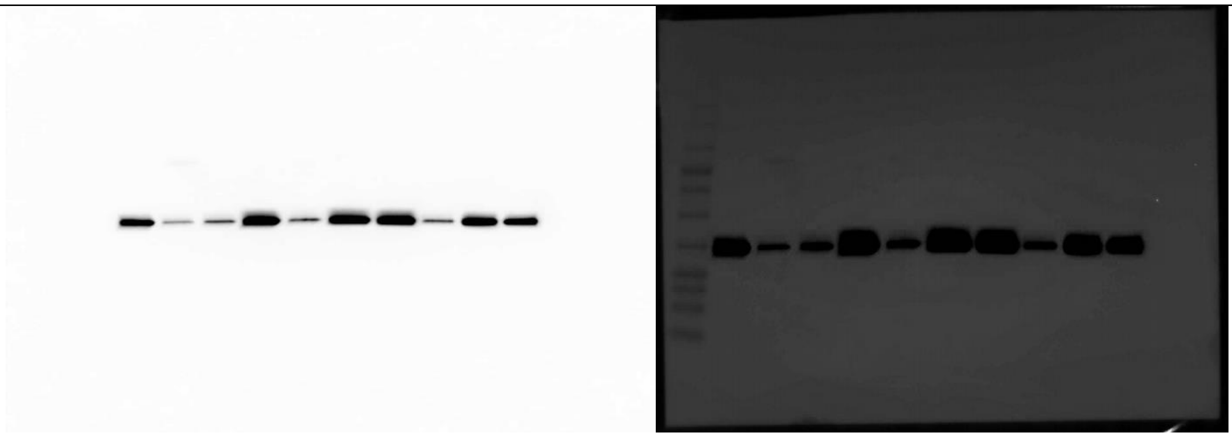
Caspase-3 (retrieved from 10sec exposure)	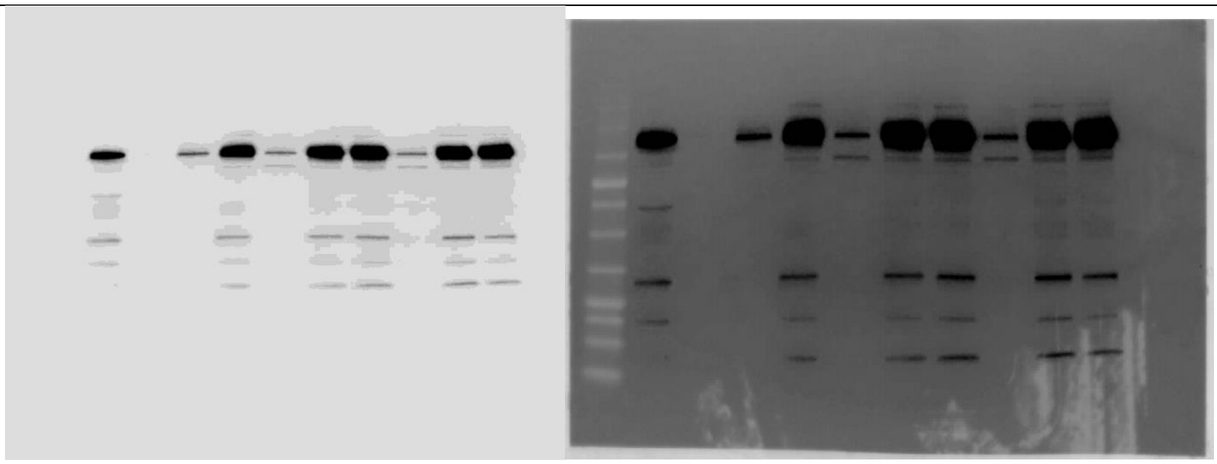
PARP (retrieved from 3sec exposure)	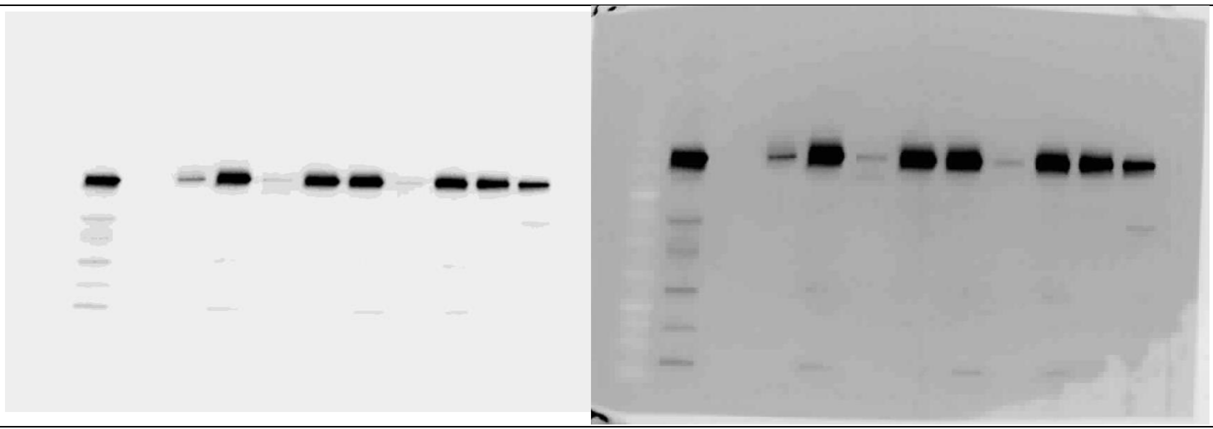

UF-extraction

Well Order	Ladder-Control-A100-A12.5-A6.25-E100-E12.5-E1.56-M100-M12.5-M3.12<br>(A indicates Acetonic. E indicates Ethanolic. M indicates Methanolic)
Beta-aktin (retrieved from 1sec exposure)	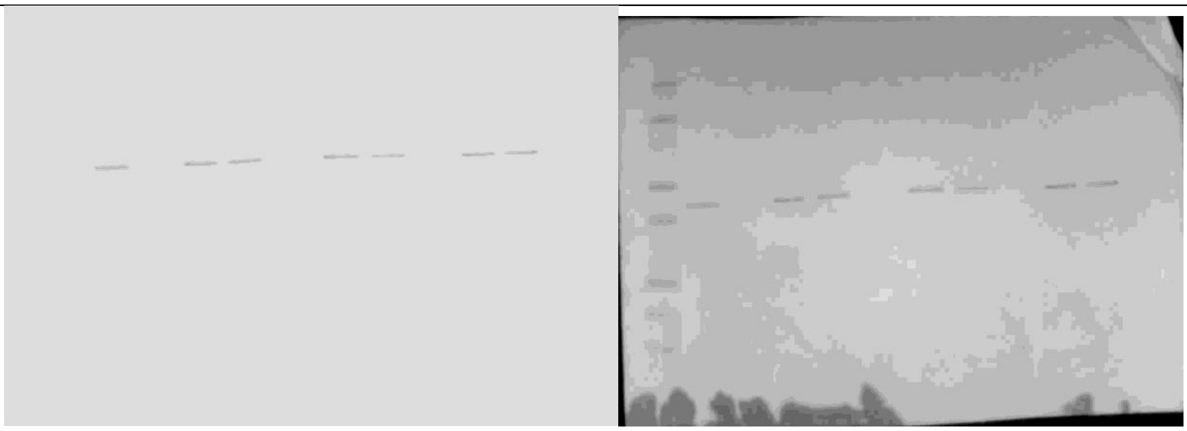
Beta-aktin (retrieved from 15sec exposure)	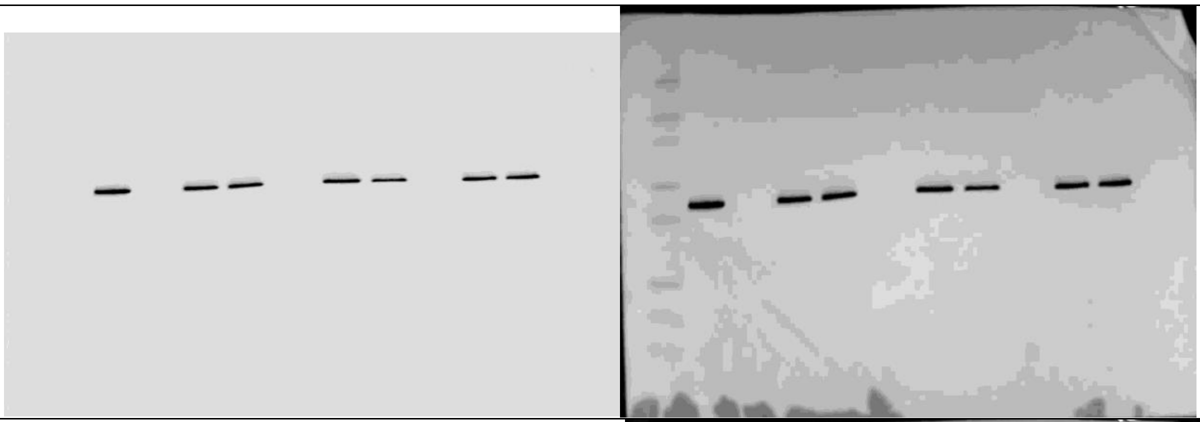
Beta-aktin (retrieved from 30sec exposure)	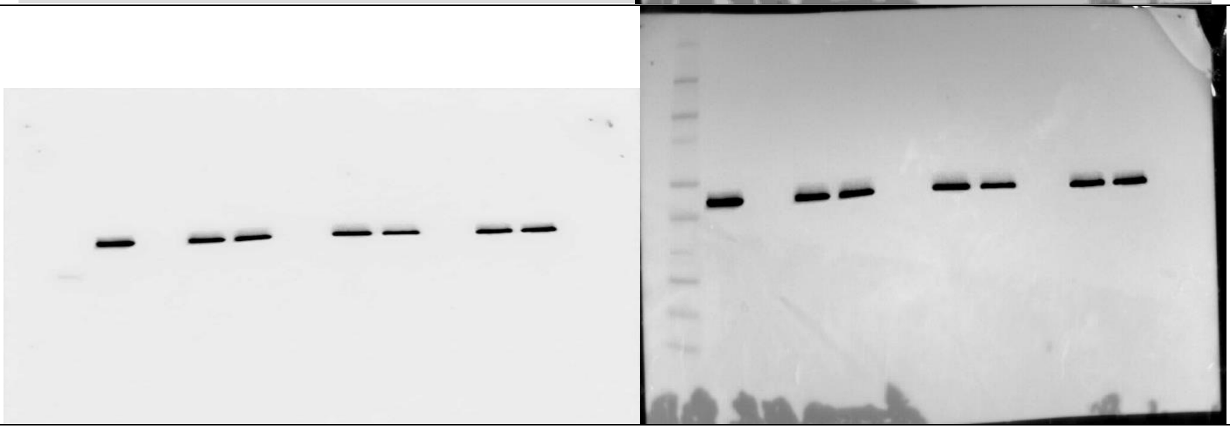

PCNA (retrieved from 1sec exposure)	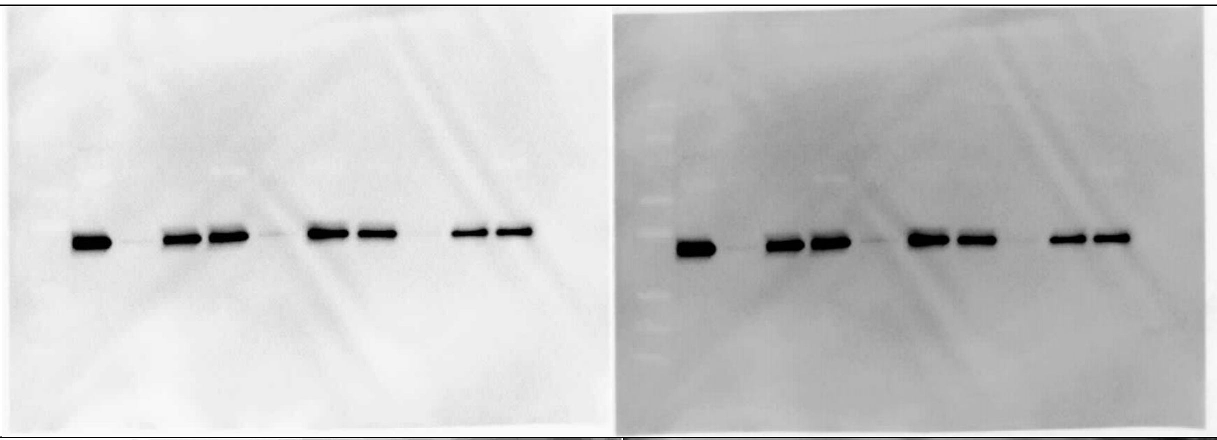
Caspase-3 (retrieved from 30sec exposure)	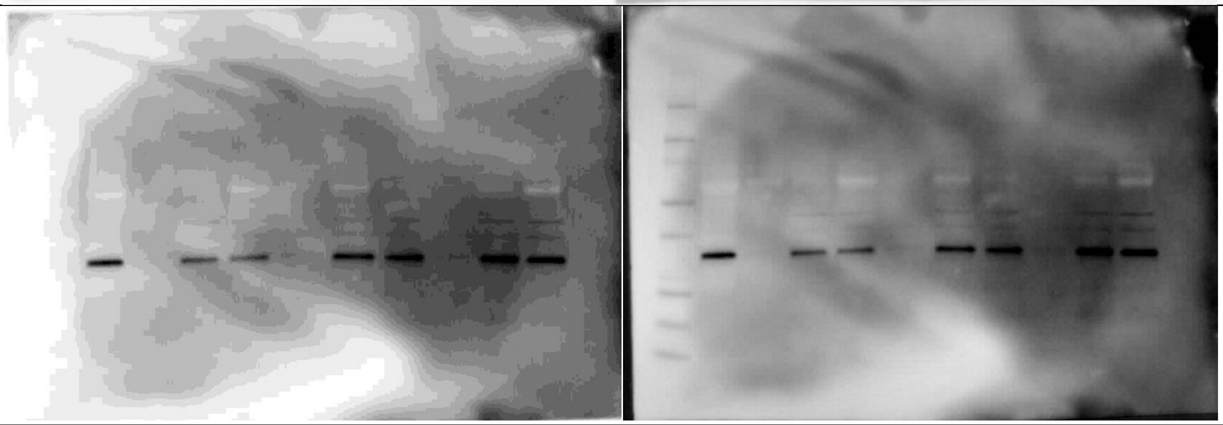
PARP (retrieved from 30sec exposure)	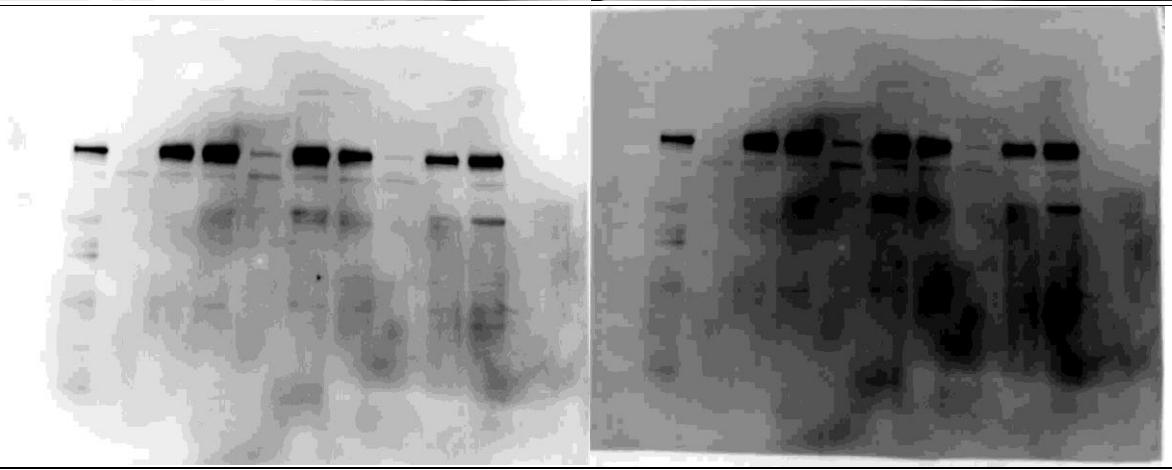
