# Supplementary material for: Differential anti-proliferative and apoptotic effects of lichen species on human prostate carcinoma cells
Source: PLoS One. 2020 Sep 30;15(9):e0238303. doi: 10.1371/journal.pone.0238303 (PMC7527208; doi:10.1371/journal.pone.0238303)
Supplement: S2 File — (PDF) [file pone.0238303.s002.pdf]

Bryoria capillaris

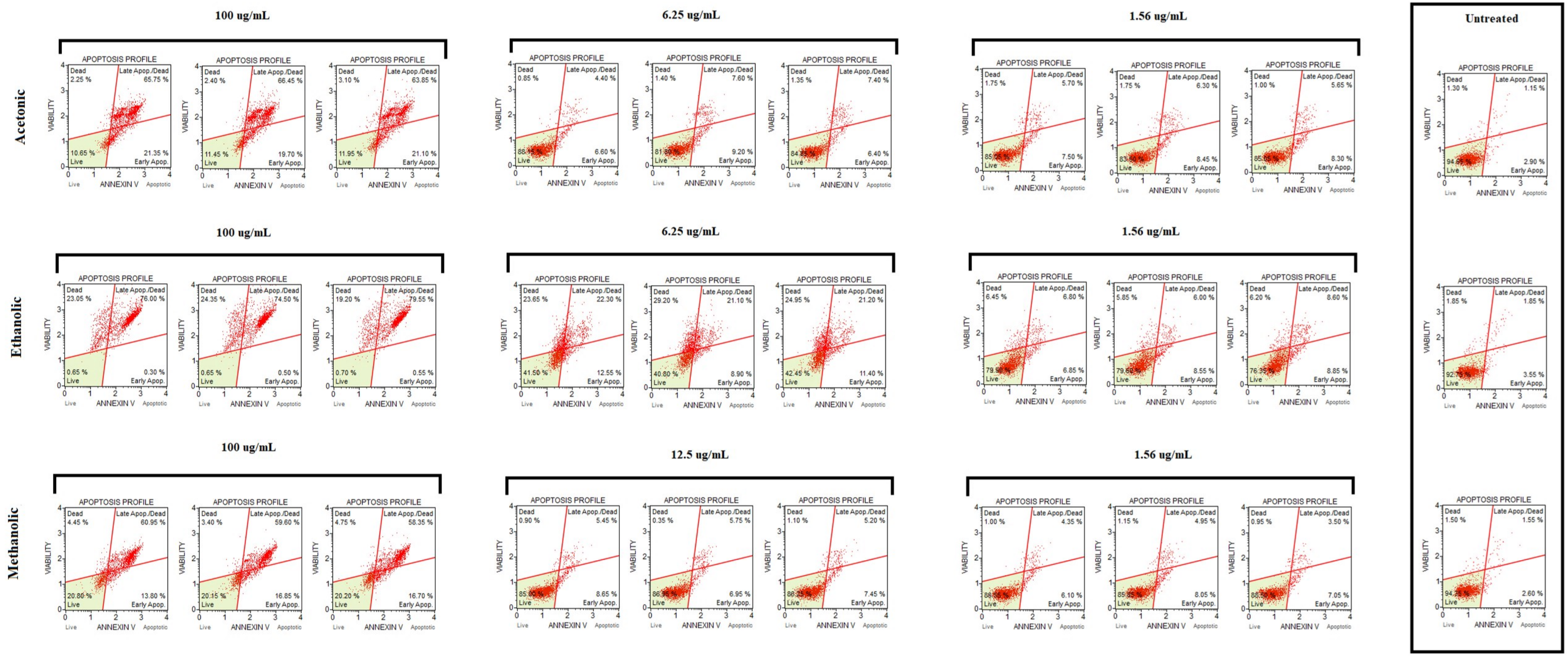

Cladonia fimbriata

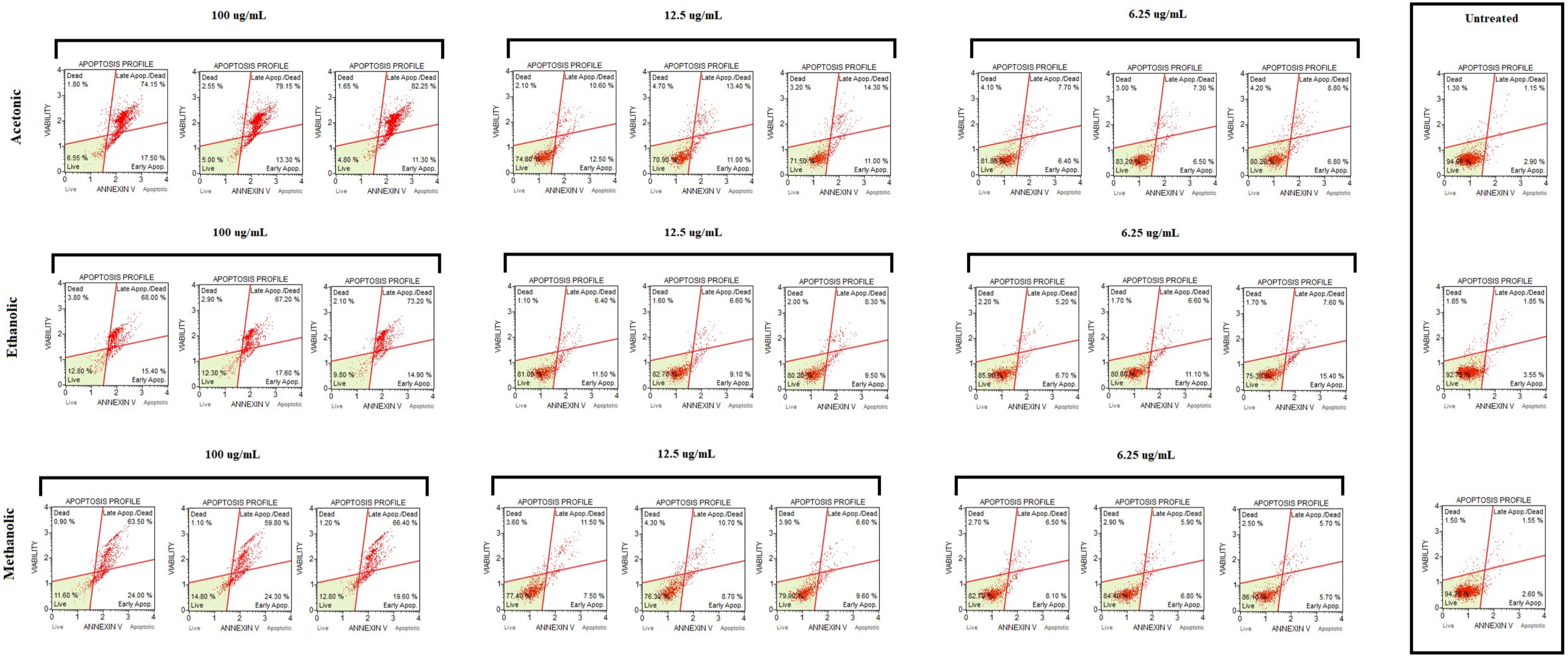

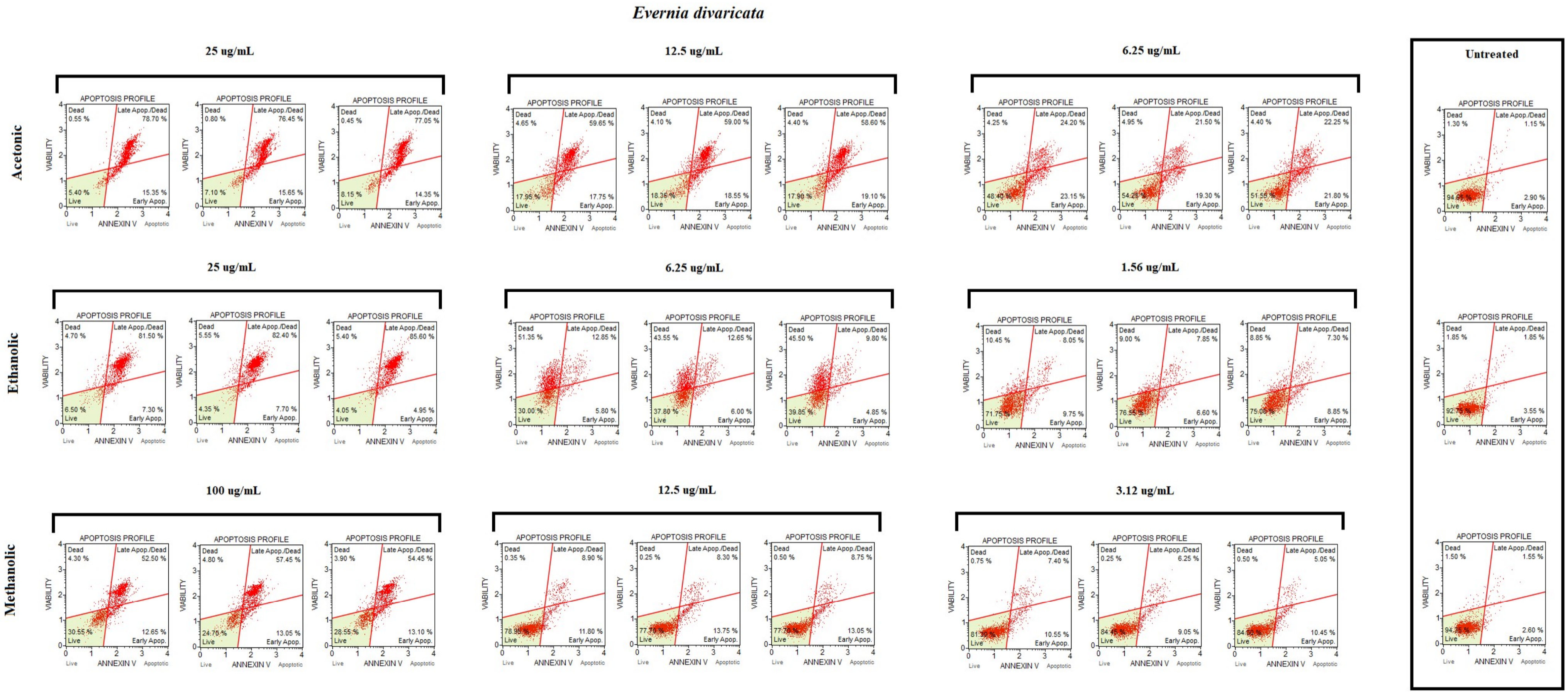

Evernia divaricata
25 ug/mL
12.5 ug/mL
6.25 ug/mL
Untreated
Acetonic
25 ug/mL
6.25 ug/mL
1.56 ug/mL
Ethanolic
100 ug/mL
12.5 ug/mL
3.12 ug/mL
Methanolic
APOPTOSIS PROFILE
Dead
Late Apop./Dead
Live
Early Apop.
VIABILITY
ANNEXIN V
Apoptotic

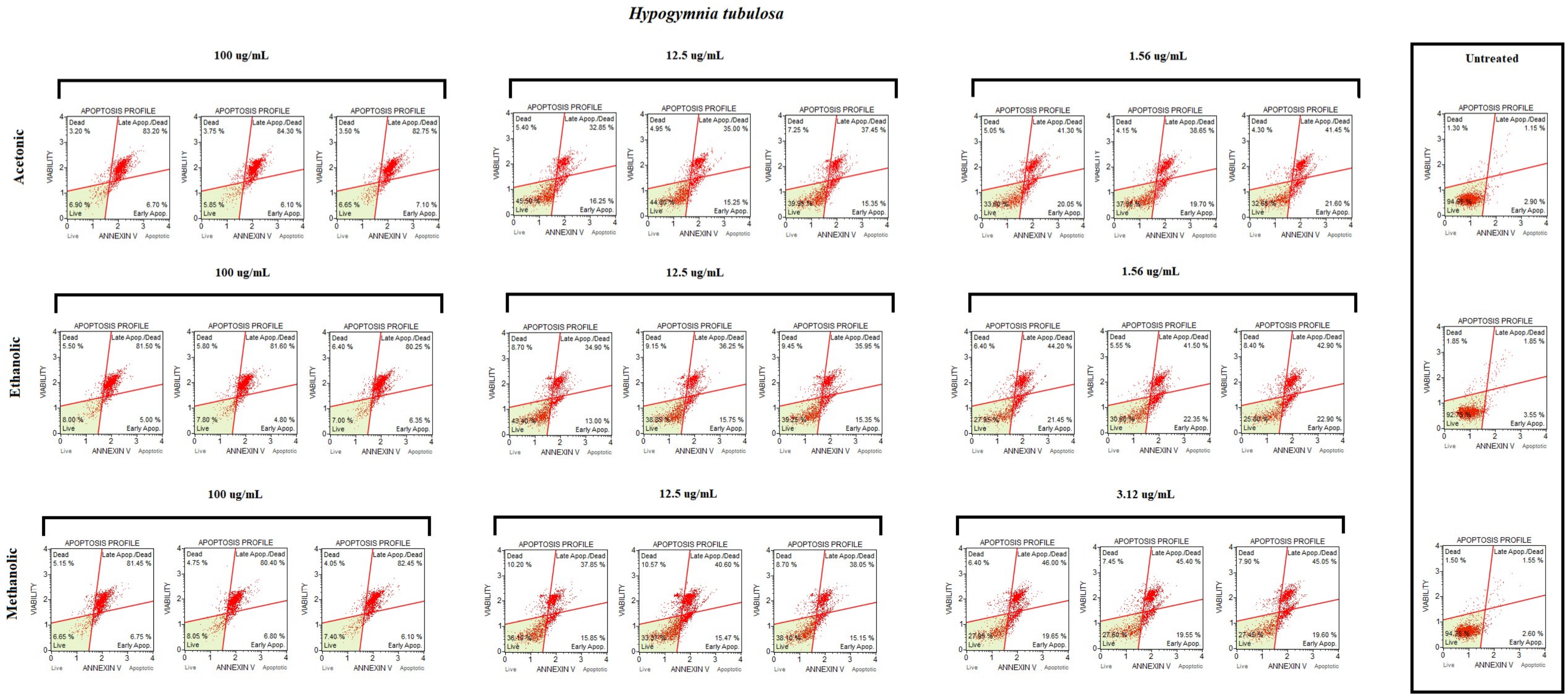
Hypogymnia tubulosa
100 ug/mL
12.5 ug/mL
1.56 ug/mL
3.12 ug/mL
Untreated
Acetonic
Ethanolic
Methanolic
APOPTOSIS PROFILE
Dead
Late Apop./Dead
Live
Early Apop.
VIABILITY
ANNEXIN V
Apoptotic

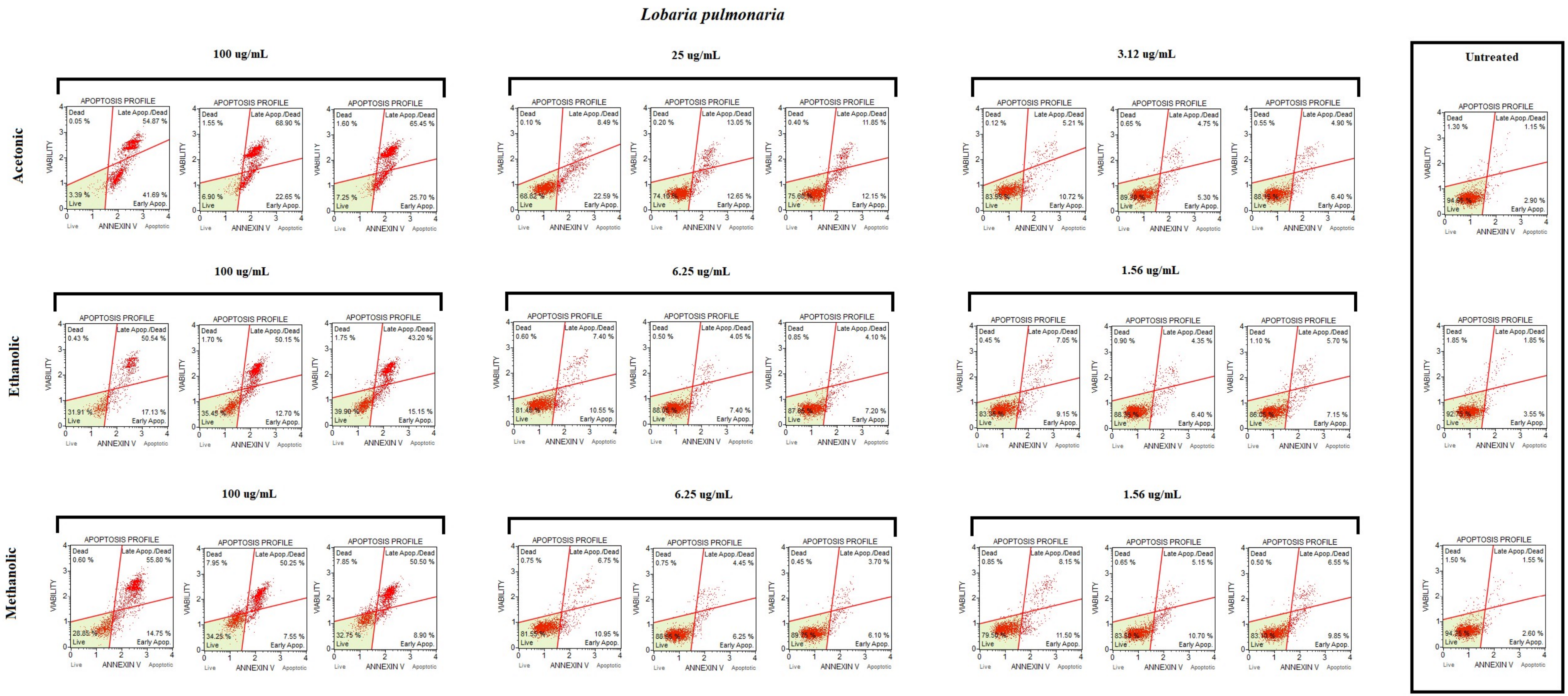
Lobaria pulmonaria
100 ug/mL
25 ug/mL
3.12 ug/mL
Untreated
Acetonic
100 ug/mL
6.25 ug/mL
1.56 ug/mL
Ethanolic
100 ug/mL
6.25 ug/mL
1.56 ug/mL
Methanolic
APOPTOSIS PROFILE
VIABILITY
ANNEXIN V
Dead
Late Apop./Dead
Live
Early Apop.

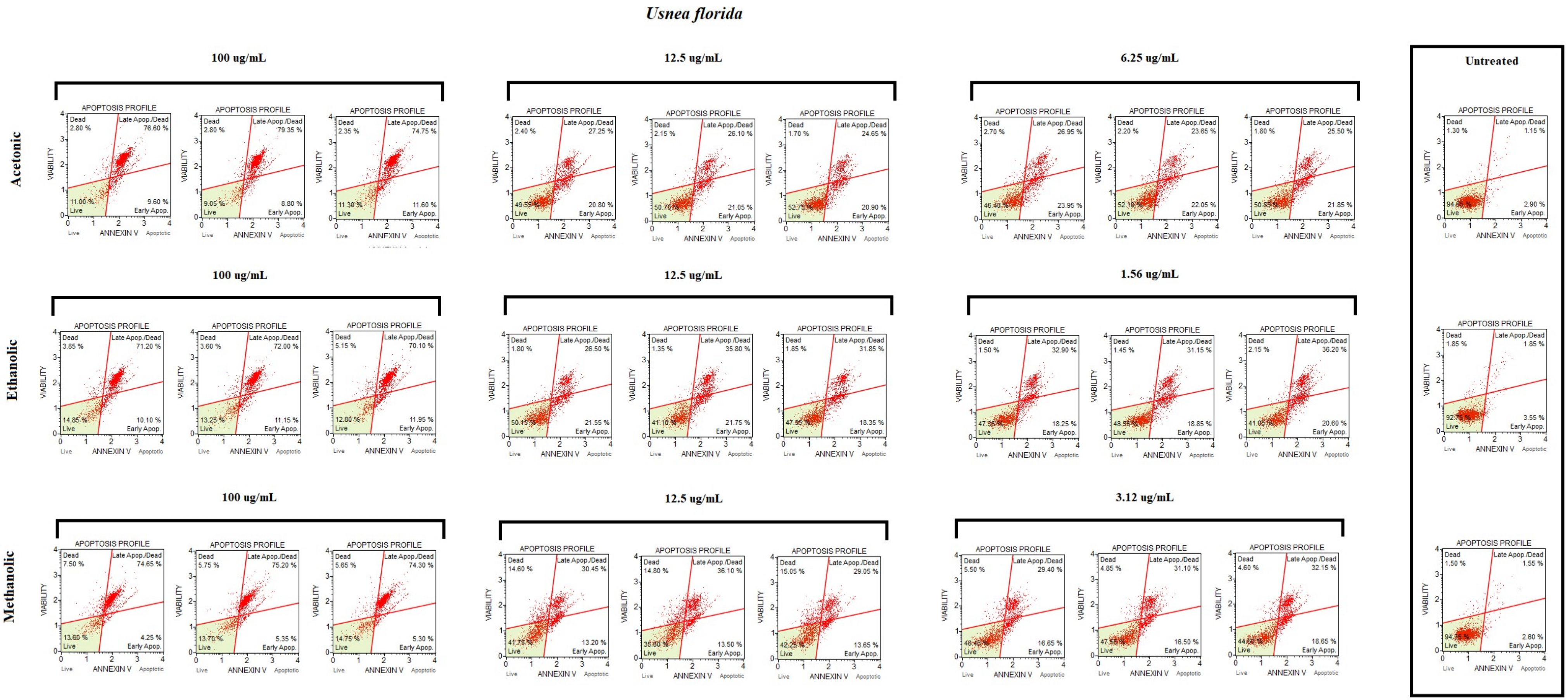
Usnea florida
100 ug/mL
12.5 ug/mL
6.25 ug/mL
Untreated
Acetonic
100 ug/mL
12.5 ug/mL
1.56 ug/mL
Ethanolic
100 ug/mL
12.5 ug/mL
3.12 ug/mL
Methanolic
APOPTOSIS PROFILE
VIABILITY
ANNEXIN V
Dead
Late Apop./Dead
Live
Early Apop.
Apoptotic
